# Distinct O-Linked Glycosylation Systems in Signaling and Immune Regulation

**DOI:** 10.3390/ijms27115119

**Published:** 2026-06-05

**Authors:** Shuguang Wang, Shibo Xiao, Yuman Huang, Xianwang Wang

**Affiliations:** Health Science Center, Yangtze University, Jingzhou 434023, China; sg.wang.stu@yangtzeu.edu.cn (S.W.); 2025711049@yangtzeu.edu.cn (S.X.); 2025711040@yangtzeu.edu.cn (Y.H.)

**Keywords:** O-glycosylation, cell signaling, immune regulation, glycoproteomics, disease pathogenesis

## Abstract

O-linked glycosylation comprises distinct regulatory systems, including secretory-pathway mucin-type O-GalNAc glycosylation and intracellular O-GlcNAcylation. These modifications both target serine/threonine residues but differ in glycan structure, cellular compartment, enzymatic machinery, and biological function. This narrative review was based on targeted searches of PubMed, Web of Science, and related literature using keywords related to O-glycosylation, O-GalNAc glycosylation, O-GlcNAcylation, immune regulation, cell signaling, glycoproteomics, and congenital disorders of glycosylation (CDG). We summarize evidence that mucin-type O-glycosylation regulates receptor behavior, cell adhesion, immune checkpoints, immunoglobulin function, antigen recognition, and pathogen–host interactions, whereas O-GlcNAcylation mainly modulates intracellular signaling, transcriptional control, stress responses, post-translational modification crosstalk, and innate immune pathways. We also discuss how glycosylation defects, including CDG and selected O-linked glycosylation disorders, connect genetic variation with disease phenotypes. Recent advances in site-specific glycoproteomics, O-glycoprotease-assisted workflows, LC–MS/MS-based glycopeptide analysis, and spatial or temporal profiling have improved mechanistic interpretation but still face limitations in site localization, structural resolution, and functional validation. Overall, the evidence supports the hypothesis that distinct O-linked glycosylation systems act through different molecular mechanisms but converge on signaling regulation, immune homeostasis, and disease susceptibility.

## 1. Introduction

Protein glycosylation is one of the most abundant and structurally diverse post-translational modifications in eukaryotic cells, with broad effects on protein folding, stability, trafficking, localization, and biological activity [1,2]. Among the major glycosylation pathways, N-glycosylation and O-glycosylation differ substantially in linkage chemistry, biosynthetic route, cellular compartment, and final glycan architecture. N-glycosylation is initiated in the endoplasmic reticulum (ER), where a preassembled oligosaccharide is transferred to the amide nitrogen of asparagine residues within defined consensus motifs, followed by extensive processing in the ER and Golgi apparatus. By contrast, classical O-glycosylation refers to the attachment of glycans to the hydroxyl groups of serine or threonine residues and includes multiple subtypes with distinct initiating sugars and enzymes. This distinction is important because different glycosylation systems generate different molecular structures and are associated with different biological and pathological consequences [1,2,3].

Among O-linked pathways, mucin-type O-glycosylation, also known as O-GalNAc glycosylation, is one of the best-characterized forms. It is primarily initiated in the Golgi apparatus by a large family of polypeptide N-acetylgalactosaminyltransferases (GalNAc-Ts), which transfer N-acetylgalactosamine (GalNAc) to serine or threonine residues on protein substrates. This initial GalNAc residue can then be extended into diverse O-glycan structures through the coordinated action of glycosyltransferases and glycosidases [2,4]. Therefore, mucin-type O-glycosylation should not be regarded as the simple addition of a single sugar, but rather as a biosynthetic process that can generate highly heterogeneous and context-dependent glycan chains. This structural flexibility allows O-GalNAc glycans to regulate protein conformation, proteolytic processing, receptor behavior, cell adhesion, and communication at epithelial and immune interfaces [2,4].

**Figure 1:**
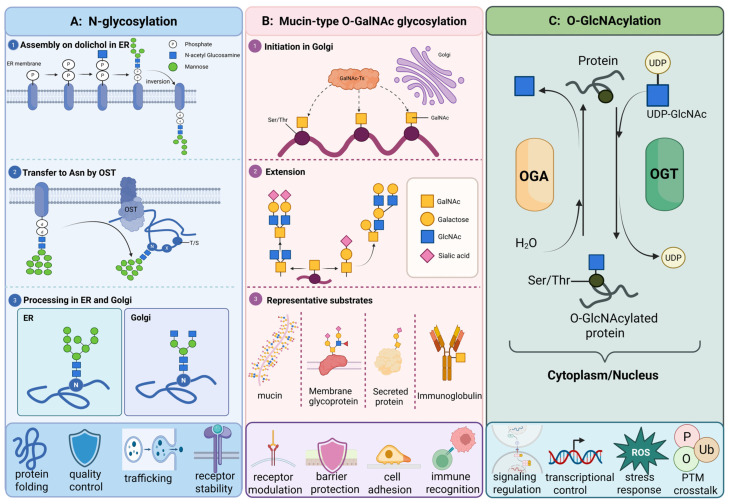
Distinct biosynthetic routes and functional outputs of N-glycosylation, mucin-type O-GalNAc glycosylation, and O-GlcNAcylation. This schematic compares three major glycosylation-related processes with distinct cellular locations, enzymatic mechanisms, glycan architectures, and biological functions. (**A**) N-glycosylation is initiated in the endoplasmic reticulum (ER), where an oligosaccharide precursor is assembled on dolichol and transferred en bloc to asparagine (Asn) residues by the oligosaccharyltransferase (OST) complex. Subsequent trimming and remodeling in the ER and Golgi apparatus generate mature N-glycans, which contribute to protein folding, quality control, intracellular trafficking, and receptor stability. (**B**) Mucin-type O-GalNAc glycosylation is initiated mainly in the Golgi apparatus by polypeptide N-acetylgalactosaminyltransferases (GalNAc-Ts), which add N-acetylgalactosamine (GalNAc) to serine or threonine (Ser/Thr) residues. The initial GalNAc residue can be further extended into diverse core structures and elongated O-glycans. This pathway is prominent in mucins, membrane glycoproteins, secreted proteins, and immunoglobulins, and regulates receptor modulation, epithelial barrier protection, cell adhesion, and immune recognition. (**C**) O-GlcNAcylation is a reversible intracellular modification occurring mainly in the cytoplasm and nucleus. O-GlcNAc transferase (OGT) uses UDP-GlcNAc to add a single N-acetylglucosamine (GlcNAc) residue to Ser/Thr residues on target proteins, whereas O-GlcNAcase (OGA) removes this modification. Unlike mucin-type O-GalNAc glycosylation, O-GlcNAcylation does not generate elongated glycan chains and primarily regulates signaling, transcriptional control, stress responses, and crosstalk with other post-translational modifications. Together, these glycosylation systems operate in different cellular compartments and regulate protein function through distinct molecular mechanisms, thereby influencing signaling regulation, immune homeostasis, disease susceptibility, cell identity and development, and therapeutic responses. Arrows indicate the direction of biosynthetic or regulatory processes; letters A–C indicate the three glycosylation systems shown in the figure; colors are used for visual distinction only. Abbreviations: Asn, asparagine; ER, endoplasmic reticulum; GalNAc, N-acetylgalactosamine; GalNAc-Ts, polypeptide N-acetylgalactosaminyltransferases; GlcNAc, N-acetylglucosamine; OGA, O-GlcNAcase; OGT, O-GlcNAc transferase; OST, oligosaccharyltransferase; PTM, post-translational modification; Ser/Thr, serine/threonine; UDP-GlcNAc, uridine diphosphate N-acetylglucosamine; UDP, uridine diphosphate; ROS, reactive oxygen species.

O-GlcNAcylation is mechanistically distinct from mucin-type O-glycosylation, although both involve modification of serine or threonine residues. O-GlcNAcylation refers to the reversible addition of a single N-acetylglucosamine (GlcNAc) residue to intracellular proteins, mainly in the cytoplasm and nucleus, by O-GlcNAc transferase (OGT), with removal by O-GlcNAcase (OGA). Unlike mucin-type O-glycans, O-GlcNAc does not undergo elongation into complex glycan chains and is not primarily a secretory-pathway modification. Instead, it often functions as a dynamic intracellular signaling modification that interacts with phosphorylation, ubiquitination, and other post-translational modifications to influence protein stability, transcriptional control, stress responses, metabolism, and immune signaling [1]. A schematic comparison of N-glycosylation, mucin-type O-GalNAc glycosylation, and O-GlcNAcylation, together with their major biological functions, is shown in Figure 1.

The biological importance of glycosylation is also underscored by congenital disorders of glycosylation (CDG), a heterogeneous group of inherited metabolic diseases caused by defects in genes involved in glycan biosynthesis, processing, transport, or attachment. CDG can affect N-linked, O-linked, lipid-linked, and glycosylphosphatidylinositol-anchor glycosylation pathways, and many CDG subtypes present with multisystem phenotypes involving the nervous system, liver, muscle, eye, skeleton, immune system, and other organs [3]. Although many CDG are caused by defects in N-glycosylation, O-linked glycosylation defects are also clinically important and are often tissue-specific. For example, dystroglycanopathies represent a well-known group of disorders involving defective O-mannosylation of α-dystroglycan, in which impaired glycosylation disrupts extracellular matrix binding and leads to a broad disease spectrum ranging from severe muscle–eye–brain phenotypes to milder limb-girdle muscular dystrophy or neurodevelopmental presentations [5]. These examples show that glycosylation defects can create direct links between genotype, altered glycan structure, and clinical phenotype.

The genetic landscape of glycosylation-related disorders further highlights the need to connect molecular mechanisms with disease phenotypes. Variants in genes encoding glycosyltransferases, glycosidases, nucleotide-sugar transporters, ER/Golgi processing enzymes, and accessory factors can produce distinct but sometimes overlapping clinical manifestations. Pathogenic variants in B3GALNT2 impair α-dystroglycan glycosylation and are associated with a broad dystroglycanopathy spectrum, although genotype–phenotype correlations remain complex [5]. ALG3-CDG, caused by variants in the ER mannosyltransferase ALG3, illustrates how defects in early N-glycan assembly can lead to severe multisystem disease with neurological, ocular, skeletal, and dysmorphic features [6]. More recently, MAN2A2-related glycosylation defects have been associated with neurodevelopmental phenotypes, autism spectrum disorder, and cognitive delay, emphasizing that even relatively specific defects in N-glycan processing may have tissue-selective consequences, especially in the central nervous system [7]. Although not all of these examples are O-glycosylation defects, they provide a broader genetic context for understanding how altered glycosylation pathways can produce clinically meaningful phenotypes.

Traditionally, mucin-type O-glycosylation has been viewed mainly as a structural feature that supports epithelial protection, mucus formation, and barrier function. However, accumulating evidence indicates that its functions extend far beyond structural decoration. Site-specific O-glycans can regulate protein conformation, receptor–ligand interactions, proteolytic processing, immune recognition, and cell–cell communication, thereby influencing signaling outputs and tissue homeostasis [2,4]. In parallel, O-GlcNAcylation has emerged as a major intracellular regulatory modification that links nutrient status, cellular stress, transcriptional regulation, and immune signaling. Advances in glycoproteomics, site-specific glycopeptide analysis, and functional glycoengineering have greatly expanded the known landscape of glycosylated proteins and reinforced the view that glycosylation is a widespread and functionally important regulatory layer rather than a passive surface modification [2,8].

Aberrant O-linked glycosylation has been increasingly linked to cancer, autoimmune disease, infection, inflammatory disorders, and inherited glycosylation defects. Nevertheless, many studies remain either pathway-specific or disease-specific. Mucin-type O-GalNAc glycosylation is often discussed in the context of epithelial surfaces, mucins, immunoglobulins, and cell-surface receptors, whereas O-GlcNAcylation is often discussed separately in relation to intracellular signaling and stress responses. This separation is conceptually necessary, but it can also obscure the broader question of how distinct glycosylation systems converge on common biological outcomes, including altered receptor signaling, immune checkpoint regulation, antigen recognition, inflammatory thresholds, and disease susceptibility. Therefore, a clearer framework is needed to distinguish these processes mechanistically while also explaining how they jointly shape immune regulation.

This review is based on a narrative literature search focused on O-linked glycosylation, immune regulation, cell signaling, disease mechanisms, and glycosylation-related disorders. Relevant studies were identified from PubMed, Web of Science, and related literature sources using combinations of keywords including “O-glycosylation”, “mucin-type O-glycosylation”, “O-GalNAc glycosylation”, “O-GlcNAcylation”, “immune regulation”, “cell signaling”, “Siglec”, “PD-L1”, “IgA nephropathy”, “glycoproteomics”, “congenital disorders of glycosylation”, and “CDG”. Priority was given to mechanistic studies, recent advances, and representative reviews directly related to glycosylation-dependent regulation of signaling, immunity, disease pathogenesis, and analytical technologies. This review is not intended as a systematic review, but as a mechanistic synthesis of representative evidence across related fields.

We hypothesize that distinct O-linked glycosylation systems, particularly mucin-type O-GalNAc glycosylation and O-GlcNAcylation, regulate signaling and immune responses through different molecular mechanisms, but converge functionally on immune homeostasis and disease susceptibility. Accordingly, this review first clarifies the conceptual distinction between major glycosylation pathways and then discusses how O-linked glycosylation regulates receptor signaling, cell adhesion, post-translational modification crosstalk, immune recognition, and disease-relevant cellular communication. We further consider how inherited glycosylation defects, including CDG and O-linked glycosylation-related disorders, provide genotype–phenotype evidence for the clinical importance of glycosylation pathways. Finally, we highlight analytical advances and unresolved challenges that must be addressed to move the field from association-based observation toward more precise mechanistic and translational interpretation. To provide a conceptual framework for the topics discussed in this review, Figure 1 summarizes the major biosynthetic features, cellular compartments, and representative biological functions of N-glycosylation, mucin-type O-GalNAc glycosylation, and O-GlcNAcylation.

## 2. O-Glycosylation and Cell Signaling

### 2.1. Modulation of Receptor Function

O-glycosylation represents a critical regulatory layer modulating the function of cell surface receptors. The addition of O-glycans to receptor proteins can profoundly influence receptor conformation, surface localization, ligand affinity, and subsequent activation of downstream signaling pathways. This dynamic and often site-specific modification offers cells a means to fine-tune signal transduction in response to developmental cues, physiological changes, and disease states [2].

#### 2.1.1. O-Glycans on Cell Surface Receptors

A variety of cell-surface receptors are regulated by O-glycosylation. Rather than acting as passive surface decorations, O-glycans can reshape receptor behavior by altering ectodomain organization, stabilizing protein conformation, or affecting receptor retention at the plasma membrane.

For EGFR, available evidence indicates that mucin-type O-glycosylation can influence downstream signaling output. In glioblastoma, C1GALT1-mediated O-glycan elongation is associated with altered EGFR-dependent AKT/ERK signaling, supporting the view that receptor-associated O-glycosylation can bias proliferative signaling cascades rather than merely changing surface decoration [9]. Consistently, O-GalNAc glycosylation has also been linked to regulation of EGFR-driven transcriptional programs, further suggesting that glycan remodeling can influence both proximal receptor activity and downstream cellular responses [10].

Another example is CD320. O-glycosylation has been shown to be required for efficient cell-surface expression of the transcobalamin receptor CD320, indicating that O-glycans may determine whether a receptor is properly displayed at the plasma membrane and therefore available for ligand-dependent function [11].

Notch represents a particularly informative model because it provides one of the clearest mechanistic examples of how distinct forms of O-glycosylation directly regulate receptor–ligand interaction and signaling output [12,13].

#### 2.1.2. Effects on Ligand Binding, Receptor Activation, and Downstream Signaling

O-glycosylation can regulate receptor function at multiple levels. First, glycans may alter ligand binding through steric effects, electrostatic properties, or local conformational changes at or near the binding interface. Second, O-glycans can stabilize receptor conformations that favor productive signaling. Third, by affecting receptor folding, trafficking, or surface residence, O-glycosylation can indirectly shape the intensity and duration of downstream pathway activation.

A representative host-system example is the interaction between the A1 domain of von Willebrand factor and platelet GPIbα. In this system, O-linked glycans in the vWF A1 autoinhibitory module contribute to shielding of the GPIbα-binding region. Their effects are thought to involve both steric hindrance and charge-related influences associated with sialylated residues, thereby reducing receptor engagement under force-dependent conditions [14]. This example illustrates a general principle in receptor biology: O-glycans can tune ligand accessibility in ways that become especially important in dynamic physiological environments.

In addition, receptor-associated O-glycosylation may influence downstream signaling even when the glycan does not form part of the canonical ligand-binding interface itself. As shown in EGFR-related studies, changes in O-glycan processing can be coupled to altered activation of intracellular signaling pathways such as AKT and ERK [9]. Thus, the functional consequences of O-glycosylation extend from ligand engagement to broader signaling output.

#### 2.1.3. Case Study: Notch Signaling and O-Fucosylation/O-Glucosylation

Notch signaling plays central roles in cell fate determination, tissue development, and homeostasis, and therefore serves as an ideal model for understanding receptor regulation by O-glycosylation. The extracellular domain of Notch contains multiple EGF-like repeats that can carry several forms of O-linked glycans, including O-fucose and O-glucose. These modifications are not merely decorative; they directly affect receptor folding, trafficking, ligand recognition, and signaling strength [13,15].

- O-fucosylation and ligand recognition

O-fucose is added to defined EGF repeats by POFUT1 and is crucial for efficient Notch function. Structural and functional studies have shown that specific O-fucose residues in the ligand-binding region of NOTCH1 directly contribute to interactions with Delta-like and Jagged ligands. When these glycosylated sites are altered, ligand binding and signal activation are markedly reduced, indicating that O-fucose participates directly in receptor–ligand recognition rather than serving only as a structural mark [12,13].

- Fringe-mediated extension of O-fucose

The function of O-fucose can be further refined by Fringe enzymes, which extend this modification and thereby alter ligand selectivity. This additional glycan processing does not affect all ligands equally. Instead, it tunes the responsiveness of Notch to Delta-like versus Jagged ligands in a site-dependent manner, providing a mechanism by which glycan elaboration adjusts signaling bias during development and tissue regulation [16,17]. In this sense, O-fucosylation and its extension act as part of a molecular code that helps determine how Notch interprets different extracellular inputs.

- Chemical perturbation evidence

Support for the functional importance of O-fucose also comes from chemical perturbation studies. Fucose analogs can be incorporated into Notch EGF repeats and selectively interfere with Delta-induced Notch activation, reinforcing the conclusion that this glycan modification is mechanistically involved in ligand-triggered signaling rather than merely correlated with it [18].

- O-glucosylation and receptor competence

O-glucose modification, catalyzed by POGLUT1, represents another critical regulatory layer on Notch receptors. This modification contributes to structural stability of EGF repeats and supports proper receptor maturation and signaling competence. Evidence from structural and disease-related studies indicates that disruption of POGLUT1-dependent O-glucosylation impairs Notch signaling and is associated with pathological phenotypes, including reduced satellite-cell maintenance and muscular dystrophy-related defects [19,20,21].

Taken together, Notch provides a well-defined example of how different forms of O-glycosylation cooperate to regulate receptor behavior at multiple levels. O-fucose helps shape ligand recognition, Fringe-dependent extension refines ligand selectivity, and O-glucose supports receptor structural competence. This layered regulation illustrates why O-glycosylation should be viewed as an active determinant of receptor function rather than a secondary structural feature.

### 2.2. Regulation of Cell Adhesion and Migration

O-glycosylation shapes cell adhesion and migration by remodeling the glycocalyx and the interaction interfaces of adhesion receptors and their ligands. Because O-glycans are installed in a site-, cell type-, and context-dependent manner, they can tune adhesion strength, receptor accessibility, and mechanotransduction without requiring changes in protein abundance, thereby influencing leukocyte trafficking, epithelial organization, and tissue remodeling [2].

#### 2.2.1. O-Glycans in Selectins, Cadherins, and Mucins

- Selectins

Selectin-dependent adhesion relies heavily on glycan recognition. In particular, many selectin ligands are membrane glycoproteins carrying sialylated and fucosylated determinants on O-glycan scaffolds. These O-glycans do not merely provide passive structural support; rather, they enable the multivalent display of functional glycan epitopes and thereby facilitate the transient adhesive interactions required for leukocyte tethering, rolling, and subsequent transmigration [4]. From a broader biosynthetic perspective, the diversity of O-glycan initiation and elongation pathways provides a mechanism by which cells can tune the availability of selectin-binding motifs under different physiological conditions [22].

- Cadherin

Cadherin-mediated cell adhesion can be influenced by O-glycosylation through both direct and indirect mechanisms. Directly, O-linked glycan modifications may affect the extracellular organization and adhesive behavior of cadherin-family proteins, thereby influencing intercellular junction stability [2]. Indirectly, O-glycosylation can modulate signaling pathways that determine the expression or maintenance of cadherin-based junctions. A representative example is the GALNT8–EGFR axis, in which altered O-GalNAc glycosylation of EGFR is associated with changes in downstream signaling and E-cadherin expression, ultimately affecting migratory behavior [10]. This type of regulation suggests that O-glycosylation can influence cell adhesion not only at the level of adhesion molecules themselves, but also through upstream signaling networks that stabilize or weaken epithelial identity [23].

- Mucins

Mucins are among the most extensively O-glycosylated proteins on epithelial surfaces and make major contributions to the physical properties of the glycocalyx. Their dense O-glycan chains create extended, hydrated structures that influence cell–cell and cell–matrix interactions by modifying steric spacing, surface charge, and molecular accessibility [4]. In addition to serving as structural components, mucin-associated glycans help organize the epithelial interface with the surrounding environment, thereby contributing to the regulation of adhesion-related surface properties [24]. Because mucin glycoforms are dynamically shaped by the underlying O-glycosylation machinery, changes in mucin glycosylation can alter local adhesive conditions and affect how cells move within or across tissue barriers [2].

#### 2.2.2. Functional Consequences for Cell Migration

The effects of O-glycosylation on adhesion are closely linked to cell migration. Migration requires continuous coordination between attachment and detachment, and O-glycans are well positioned to modulate this balance by altering receptor engagement, membrane organization, and the stability of adhesive contacts [1]. When glycan composition is changed, the consequences may include altered cell-surface retention of adhesion molecules, modified responsiveness to external ligands, and shifts in epithelial versus motile cellular states.

This regulatory principle is particularly evident in signaling pathways connected to adhesion remodeling. Changes in mucin-type O-glycosylation have been linked to altered EGFR signaling and downstream transcriptional programs that are relevant to cell motility [10]. Likewise, O-glycosylation-dependent control of receptor availability at the plasma membrane, as shown for CD320, supports the broader concept that glycan modification can regulate whether membrane proteins are properly displayed to participate in cell–environment interactions [11]. Together, these observations indicate that O-glycosylation contributes to migration not only by modifying classical adhesion ligands, but also by shaping the receptor systems and signaling programs that determine adhesive behavior.

### 2.3. Crosstalk with Other Post-Translational Modifications

Post-translational modifications (PTMs) greatly expand the functional repertoire of proteins by dynamically regulating their activity, localization, stability, and interactions. Within this regulatory network, O-glycosylation is increasingly recognized as an active participant rather than an isolated modification. Its functional effects often depend not only on the glycan itself, but also on coordinated interplay with other PTMs, especially phosphorylation and, to a lesser extent, ubiquitination. Through such crosstalk, O-glycosylation can integrate metabolic state, signaling context, and protein homeostasis into a unified regulatory output [25].

#### 2.3.1. Crosstalk Between O-Glycosylation and Phosphorylation

Among all PTM interactions, the relationship between O-glycosylation and phosphorylation is the best characterized. This is particularly true for O-GlcNAcylation, a highly dynamic intracellular modification that occurs on serine and threonine residues and therefore frequently intersects with phosphorylation-dependent signaling. Rather than acting as two independent marks, O-GlcNAcylation and phosphorylation often function as a coupled regulatory system that determines protein activity, interaction preference, intracellular trafficking, and transcriptional output [26].

This crosstalk can be understood in three major forms. First, the two modifications may compete for the same or overlapping serine/threonine residues, generating a mutually exclusive switch at specific sites. Second, modification at neighboring residues may alter local steric environment or recognition by kinases and phosphatases, thereby shifting phosphorylation efficiency without direct site overlap. Third, more distant O-glycosylation events may induce conformational or interaction changes that indirectly reshape phosphorylation patterns elsewhere on the protein [26].

Beyond substrate-level competition, O-glycosylation can also influence phosphorylation networks by acting on the enzymes that write, erase, or interpret phosphosignals. Kinases and phosphatases themselves are subject to O-GlcNAcylation, and the enzymes responsible for O-GlcNAc cycling—OGT and OGA—are also regulated within signaling cascades. As a result, O-glycosylation can affect phosphorylation not only locally on individual proteins, but also more broadly at the pathway level [25]. This systems-level view is especially important in cell signaling, where modest changes in O-GlcNAc occupancy may propagate into larger shifts in kinase activity and downstream transcriptional programs.

Although intracellular O-GlcNAcylation provides the clearest example of direct phospho-crosstalk, secretory-pathway O-glycosylation can also influence phosphorylation-dependent signaling in a more indirect manner. By regulating receptor folding, ligand binding, membrane residence, or receptor accessibility, extracellular O-glycosylation can shape activation of downstream phosphorylation cascades even when the glycan itself is not installed near the final phosphosite [4,10]. In this sense, O-glycosylation should be viewed not simply as a parallel PTM, but as a modification capable of reorganizing phosphorylation-driven signaling at multiple levels.

#### 2.3.2. Crosstalk Between O-Glycosylation and Ubiquitination

Compared with phosphorylation, the relationship between O-glycosylation and ubiquitination is less comprehensively defined and appears to be more context-dependent. Current evidence more often supports an indirect functional connection than a universal direct modification pair. In many settings, O-glycosylation alters protein folding, conformational stability, intracellular trafficking, or signaling state, and these upstream effects subsequently influence whether a protein is retained, internalized, or targeted for ubiquitin-dependent degradation.

This principle is consistent with the broader role of O-glycosylation in protein quality control and membrane protein homeostasis. When glycosylation changes receptor maturation or surface presentation, it may secondarily alter exposure to degradation pathways, including ubiquitin-mediated turnover. Accordingly, the functional relationship between O-glycosylation and ubiquitination is often best understood as part of a larger regulatory sequence linking glycan-dependent protein handling to stability control, rather than as a simple one-step antagonistic interaction [11].

At present, the strongest general conclusion is that O-glycosylation can influence ubiquitin-dependent protein fate by changing the structural and signaling context in which ubiquitination occurs. This effect may be biologically important, but the underlying mechanisms are not yet as broadly established or as mechanistically resolved as those described for O-glycosylation–phosphorylation crosstalk [2].

## 3. O-Glycosylation in Immune Regulation

### 3.1. Siglec-Mediated Modulation of Immune Cell Activation

In immune homeostasis, cell-surface glycans are not merely passive structural decorations. Rather, they function as a readable molecular code that helps determine activation thresholds, restricts excessive immune amplification, and shapes the balance between protective immunity and tolerance. Within this framework, sialic acid-binding immunoglobulin-like lectins (Siglecs) constitute a major class of glycan-sensing receptors that translate information encoded in sialylated glycoconjugates into cellular responses. Most CD33-related Siglecs contain cytoplasmic inhibitory motifs, typically immunoreceptor tyrosine-based inhibitory motifs (ITIMs) and/or ITIM-like sequences, which recruit phosphatases such as SHP-1 and SHP-2 after receptor engagement and phosphorylation, thereby attenuating proximal activation signaling. Through this mechanism, sialylated O-glycan-bearing ligands on host cells, stromal cells, or tumor cells can impose a restraining effect on immune-cell activation and contribute to the maintenance of tissue homeostasis [27,28,29,30].

From a mechanistic perspective, the biological significance of the Siglec axis lies not simply in ligand recognition itself, but in its capacity to convert glycan engagement into inhibitory immune tuning. Siglecs recognize terminal sialic acid residues displayed on glycoproteins and glycolipids, and their binding can occur either in cis, on the same cell surface, or in trans, between opposing cells. This distinction is important because cis interactions may mask or pre-organize Siglec receptors at the cell surface, whereas trans interactions allow sialylated ligands on neighboring cells, stromal components, or tumor cells to directly modulate immune-cell behavior. In many tissues, mucin-type O-glycans and terminal sialylation provide abundant ligands for Siglec family members. This allows glycosylated proteins to serve as functional checkpoints that modulate receptor signaling intensity, cytokine production, cytotoxicity, and antigen responsiveness. In pathological settings—especially cancer—this physiological glycan-dependent regulatory system is frequently co-opted to establish an immunosuppressive microenvironment [27,28,30].

In T cells, Siglec-mediated glycan recognition can directly lower the strength of activation signals and thereby restrain sustained effector responses. Although Siglecs are more commonly discussed in myeloid cells, B cells, and NK cells, several Siglec family members are also functionally relevant in T-cell regulation. Under pathological conditions, including cancer, Siglec expression or ligand availability can increase on activated or dysfunctional T-cell subsets. After engagement by sialylated ligands, inhibitory Siglecs undergo tyrosine phosphorylation within ITIM or ITIM-like motifs and recruit SHP-1/SHP-2 phosphatases. These phosphatases can counteract proximal T-cell receptor signaling and reduce downstream activation programs, including cytokine secretion and cytotoxic effector function. In this way, Siglec-mediated recognition provides a glycan-dependent inhibitory layer that operates alongside classical protein-based checkpoint pathways such as PD-1/PD-L1 [27,28,30].

A representative example is Siglec-7, which has traditionally been viewed as a receptor associated with innate immune cells but is now also recognized as functionally relevant in T-cell regulation. Siglec-7 can interact with ligands displayed on activated T cells and modulate T-cell behavior in a suppressive manner. Experimental evidence indicates that interference with Siglec-7 signaling enhances T-cell activation in co-culture systems and increases the production of inflammatory cytokines such as IFN-γ and IL-6, supporting the view that Siglec-7 can function as an inhibitory glyco-immune checkpoint in the immune synapse. These findings are important because they place glycan-dependent recognition alongside classical protein-based checkpoint systems as a determinant of T-cell responsiveness [30,31].

Siglec-9 and other Siglec family members may further contribute to T-cell suppression through both direct and indirect mechanisms. On the one hand, sialylated ligands in the tumor microenvironment may engage Siglecs on T cells and reduce their activation threshold. On the other hand, T-cell suppression can also emerge indirectly through the influence of Siglec-expressing myeloid populations. In glioblastoma, for example, Siglec-9 has been identified as an immune checkpoint molecule on macrophages, where it restricts effective T-cell priming and weakens antitumor immune responses. This illustrates that the Siglec axis does not act only in a cell-autonomous fashion; instead, it may shape T-cell activity through multicellular immune circuits in which glycan-recognition events in myeloid cells feed back onto lymphocyte activation states [27,32].

In NK cells, Siglec-mediated inhibition is even more clearly established. Siglec-7 and Siglec-9 are among the best-characterized inhibitory receptors involved in restraining NK-cell cytotoxicity. Their engagement by hypersialylated ligands on tumor cells suppresses degranulation and cytokine release, thereby weakening immune-cell killing. Studies in prostate cancer have shown that sialylated glycoproteins on tumor cells can engage Siglec-7 and Siglec-9 and markedly inhibit immune-cell cytotoxicity. This provides a direct example of how aberrant glycosylation on malignant cells can be translated into a functional immune-escape signal through the Siglec pathway [33]. Similarly, in hepatocellular carcinoma, enhanced Siglec-9–Siglec-9 ligand interactions on NK cells are associated with impaired NK-cell effector function and poorer clinical outcome, further supporting the idea that glycan-dependent inhibitory signaling dampens antitumor immunity by actively constraining innate cytotoxic programs [34].

The suppressive effect of Siglecs on NK cells should not be viewed merely as a binary on/off mechanism. Rather, it reflects a broader reprogramming of the NK-cell activation threshold. Once Siglec receptors engage sialylated ligands, inhibitory phosphatase recruitment counteracts activating receptor cascades, leading to reduced cytolytic granule release, lower IFN-γ production, and decreased killing efficiency. In physiological settings, such regulation may help prevent excessive damage to normal tissues that display endogenous sialylated glycans. In tumors, however, this same mechanism is frequently exploited by malignant cells through hypersialylation, effectively converting a homeostatic safety system into a shield against immune elimination [28,29,30].

In B cells, glycan-dependent inhibitory signaling is classically represented by CD22, a B-cell-restricted Siglec that negatively regulates B-cell receptor signaling. Although much of the translational literature around CD22 has focused on CAR-T therapy and related intervention strategies, its more fundamental biological role lies in maintaining signaling restraint and peripheral tolerance. By recognizing sialylated ligands and transducing inhibitory signals, CD22 helps prevent excessive B-cell activation and contributes to the control of antibody responses. This places the Siglec axis within the broader architecture of self-tolerance, where glycan recognition operates as a complementary regulatory layer alongside canonical antigen-receptor signaling checkpoints [28].

A related example of Siglec-linked suppression is seen in chronic lymphocytic leukemia, where T-cell dysfunction is mediated in part by Siglec-10 ligands such as CD24 and CD52 expressed on leukemia cells. In this setting, the glycan-dependent inhibitory pathway contributes to defective T-cell activation, impaired proliferation, and functional exhaustion. Although this example crosses the boundary between lymphocyte subsets, it is mechanistically informative because it shows how Siglec–ligand interactions can help establish a suppressive immune niche that secondarily alters adaptive immunity [35].

In myeloid cells, the Siglec axis extends beyond simple inhibition of activation and contributes to the remodeling of inflammatory phenotypes. Siglec-5, Siglec-7, Siglec-9, and Siglec-10 are expressed on monocytes, macrophages, and related myeloid populations, where they can suppress phagocytosis, attenuate inflammatory signaling, and shape cytokine output. Particularly relevant is the observation that Siglec-9 engagement can support immunosuppressive macrophage states. In tumor settings, Siglec-9-associated signaling has been linked to macrophage polarization toward phenotypes that are more compatible with immune suppression and tumor progression [32]. In parallel, tumor-derived mucin-type glycoproteins may participate in these processes by providing ligands or glycan environments that favor inhibitory lectin signaling. This is consistent with evidence that glycan-rich tumor ligands can bias macrophage behavior toward suppressive states, thereby reinforcing a tissue microenvironment unfavorable for effective antitumor immunity [27,30].

Taken together, these observations indicate that Siglecs should be understood as central components of a glycan-dependent immune regulatory network rather than as isolated receptors acting in individual cell types. In T cells, they constrain activation and inflammatory output; in NK cells, they reduce cytotoxicity and cytokine secretion; in B cells, they help maintain signaling restraint and peripheral tolerance; and in myeloid cells, they shape antigen presentation, inflammatory tone, and macrophage polarization. What unifies these distinct effects is the fact that sialylated glycan ligands—many of them carried on heavily O-glycosylated surface proteins—serve as upstream molecular cues that tune the intensity and quality of immune responses. Thus, the Siglec axis provides a mechanistic link between O-glycan presentation and the establishment of immune thresholds in both physiological homeostasis and pathological immune suppression [27,28].

### 3.2. Impact of O-Glycosylation on the PD-1/PD-L1 Checkpoint

The regulation of the PD-1/PD-L1 checkpoint by O-glycosylation can be understood at two interconnected levels: direct effects on checkpoint protein stability and indirect effects mediated through the tumor immune microenvironment. This distinction is important because it separates molecular events occurring on checkpoint proteins such as PD-L1 from broader glycosylation-dependent mechanisms that regulate immune-cell recruitment, cytokine production, and antitumor immune activation.

At the direct checkpoint level, O-GalNAc glycosylation contributes to the stability and surface maintenance of PD-L1. GALNT6, an O-GalNAc transferase, has been shown to interact with PD-L1 and promote its O-glycosylation in pancreatic ductal adenocarcinoma cells. When GALNT6 is knocked down, PD-L1 O-glycosylation is reduced, its ubiquitination is increased, and its proteasome-dependent degradation is accelerated, leading to lower PD-L1 abundance. This reduction is accompanied by diminished PD-1 expression on co-cultured T cells and enhanced tumor-cell susceptibility to T-cell-mediated killing, indicating that O-GalNAc glycosylation can strengthen checkpoint-mediated immune evasion by stabilizing PD-L1 at the protein level [36].

Importantly, GALNT6 also regulates antitumor immunity through a PD-L1-independent innate immune pathway involving STING. STING is a central adaptor of cytosolic DNA sensing and promotes downstream TBK1/IRF3 activation, type I interferon production, and chemokine release. In pancreatic ductal adenocarcinoma models, GALNT6 knockdown increases STING protein abundance and TBK1 phosphorylation, leading to enhanced production of IFN-β, CXCL10, and CCL5. These cytokines are important for immune-cell recruitment and activation, particularly cytotoxic CD8+ T-cell infiltration and macrophage-mediated antitumor responses. Functionally, the enhanced T-cell cytotoxicity and macrophage phagocytosis observed after GALNT6 knockdown can be reduced by STING inhibition, supporting the view that the immune-restorative effect of GALNT6 loss is at least partly STING dependent [36].

Mechanistically, the GALNT6–STING axis appears to operate through a non-classical route. GALNT6 knockdown does not primarily activate STING through increased DNA damage, mitochondrial DNA release, or cGAMP production. Instead, available evidence suggests that GALNT6-mediated glycosylation restrains STING trafficking and promotes its degradation. When GALNT6 is reduced, STING shows decreased glycosylation, improved movement from the ER to the Golgi, and reduced lysosome-dependent degradation. This allows STING to accumulate in the Golgi compartment and sustain downstream immune signaling. Therefore, GALNT6 can suppress innate antitumor immunity by limiting STING activation and reducing the production of immune-recruiting cytokines [36].

These findings indicate that GALNT6 has a dual immunosuppressive function in pancreatic cancer. On the one hand, GALNT6 stabilizes PD-L1 by reducing ubiquitin-proteasome-mediated degradation, thereby enhancing PD-1/PD-L1 checkpoint signaling. On the other hand, GALNT6 suppresses STING-dependent innate immune activation, thereby reducing IFN-β, CXCL10, and CCL5 production and weakening immune-cell infiltration. This dual mechanism is conceptually important because it shows that mucin-type O-GalNAc glycosylation can regulate tumor immune evasion at both the checkpoint-protein level and the innate immune signaling level [36].

In addition to GALNT6-dependent regulation, O-glycosylation can influence the PD-1/PD-L1 axis indirectly by modulating cytokine-dependent communication between tumor cells and immune cells. A representative example is provided by C1GALT1-dependent O-glycosylation of IL-6. In head and neck cancer models, C1GALT1-mediated O-glycosylation at threonine 166 stabilizes IL-6 and prevents its degradation, thereby sustaining IL-6/STAT3 signaling. Loss of C1GALT1 reduces IL-6 output, shifts macrophages away from an immunosuppressive phenotype, and enhances cytotoxic T-cell activity. Under these conditions, blockade of IL-6 signaling phenocopies the effect of impaired O-glycosylation, and its combination with anti-PD-1 treatment further improves immune-mediated tumor killing. These findings suggest that O-glycosylation may regulate the PD-1/PD-L1 pathway not only through checkpoint proteins themselves but also through cytokine networks that determine the immune context in which this checkpoint operates [37].

A more limited but still relevant question is whether glycosylation directly affects PD-1 itself. Glycoproteomic analysis of the human PD-1 extracellular domain has demonstrated that PD-1 carries structurally defined glycans, indicating that glycosylation is part of its native molecular architecture [38]. However, compared with the evidence available for PD-L1 stabilization, GALNT6-dependent STING suppression, or IL-6-mediated immune remodeling, the functional consequences of these glycan structures for PD-1 conformation, ligand engagement, or antibody recognition remain less firmly established. Therefore, at the current stage, it is more appropriate to treat PD-1 glycosylation as a potentially important structural feature rather than to overstate its mechanistic contribution to checkpoint signaling.

Taken together, current evidence indicates that O-glycosylation modulates the PD-1/PD-L1 checkpoint through both direct and indirect routes. On the one hand, it can stabilize PD-L1 and maintain its immunosuppressive function on tumor cells; on the other hand, it can reshape cytokine-dependent crosstalk among tumor cells, macrophages, and T cells, thereby altering the overall output of checkpoint signaling. This dual mode of regulation highlights that the influence of O-glycosylation on immune checkpoints should be interpreted not only at the level of protein modification, but also within the broader framework of glycan-controlled immune communication [36,37].

### 3.3. O-Glycosylation of Immunoglobulins

Among immunoglobulin isotypes, IgA provides the most representative example of the functional importance of O-glycosylation in immune regulation. In contrast to many other antibody molecules, human IgA1 contains a hinge region rich in serine and threonine residues that can carry multiple mucin-type O-glycans. This structural feature distinguishes IgA1 from IgA2 and gives IgA1 a greater capacity for glycan-dependent modulation of molecular conformation, receptor interaction, and immune behavior [39,40].

The functional significance of this difference begins at the structural level. IgA1 and IgA2 are not simply two subclasses with minor sequence variation; they differ in hinge-region composition, protease susceptibility, and glycosylation architecture. Because the IgA1 hinge region contains clustered O-glycosylation sites, changes in glycan occupancy or composition can alter local flexibility and molecular exposure more readily than in IgA2. This makes IgA1 particularly responsive to variation in glycosyltransferase activity and glycan processing, and therefore more likely to serve as a dynamic interface between antibody structure and immune environment [39].

This structural plasticity is reflected in substantial glycan heterogeneity. Even when glycosyltransferase expression appears comparable, IgA molecules may still display site-specific and subclass-specific glycoform differences, indicating that O-glycosylation is regulated not only by enzyme abundance but also by substrate accessibility, local protein context, and biosynthetic processing [39]. Population-level glycomic and glycoproteomic studies further suggest that IgA glycosylation is influenced by both genetic background and environmental factors, making it a dynamic rather than fixed molecular trait [40].

At the functional level, IgA O-glycosylation contributes to the maintenance of immune homeostasis in several ways. First, glycan composition affects antibody stability and structural behavior. Engineering studies on recombinant secretory IgA show that glycosylation-related molecular features influence antibody assembly and stability, supporting the view that glycans are integral to IgA persistence and function rather than peripheral additions [41]. Second, because IgA operates primarily at mucosal surfaces, its glycosylation state is closely linked to the balance between immune exclusion, microbial containment, and inflammatory restraint. In this sense, O-glycosylation helps IgA function not only as an effector molecule, but also as a regulator of mucosal equilibrium.

The homeostatic relevance of glycosylation is further supported by studies linking O-glycosylation machinery to B-cell distribution and IgA biology. Loss of GalNAc-T14 has been shown to connect defective O-glycosylation with altered B-cell homing in the context of IgA-related immune dysregulation, suggesting that glycosylation defects may affect not only antibody structure itself but also the organization of the immune compartments responsible for IgA production and trafficking [42]. Similarly, disruption of core 1 O-glycan synthesis through Cosmc deficiency impairs B-cell tolerance and promotes spontaneous autoimmunity, indicating that proper glycan biosynthesis is required for maintaining normal humoral immune control [43].

Taken together, these findings indicate that O-glycosylation of immunoglobulins, especially IgA1, should be viewed as an active determinant of immune homeostasis rather than a passive post-translational modification. By shaping antibody structure, stability, and immune context dependence, O-glycans help regulate how IgA functions at the interface between mucosal defense and immune tolerance. The pathological consequences of aberrant IgA O-glycosylation, particularly in IgA nephropathy and related autoimmune conditions, are discussed in Section 4.2 [40,42].

### 3.4. The Impact of O-Glycosylation on Antigen Recognition and Immune Tolerance

O-glycosylation can influence immune recognition not only by modifying protein structure, but also by generating glycan-dependent epitopes that alter how antigens are processed, presented, and sensed by immune receptors. In this context, the immunological significance of O-glycans lies in their ability to reshape antigenicity rather than simply decorate protein backbones. This is particularly evident for truncated mucin-type O-glycans, which can expose novel surface determinants and thereby change the interface between antigens and the immune system.

A representative example is provided by the tumor-associated carbohydrate antigens Tn and sTn. These truncated O-glycan structures are frequently exposed in malignant cells as a consequence of incomplete glycan elongation. Their appearance can generate glyco-epitopes that are absent or poorly accessible in normal tissues, making them immunologically distinctive. In this sense, abnormal O-glycosylation does not merely accompany malignant transformation, but can directly alter the antigenic landscape recognized by immune cells and antibodies [44].

The effect of O-glycosylation on antigenicity is not limited to the presence or absence of a sugar residue. Glycans can also modify local peptide conformation and thereby influence epitope presentation. Structural analysis of antibodies recognizing sTn-containing antigens has shown that immune recognition depends on a precise spatial arrangement of both the glycan and the adjacent molecular scaffold, indicating that glycan-dependent epitopes are often composite structures rather than isolated sugar motifs [44]. This helps explain why glycosylation can substantially alter antibody binding without requiring major changes in the underlying amino acid sequence.

In parallel, glycan-dependent antigen uptake can shape downstream immune responses. GalNAc-containing glycopeptides can be selectively recognized by macrophage galactose-type lectin (MGL), a C-type lectin receptor expressed on dendritic cells and other antigen-presenting cells. This recognition promotes uptake and processing of glycopeptide antigens and can enhance the resulting humoral response. A representative example is the use of GalNAc-cluster-modified MUC1 glycopeptides, which improve targeting to MGL-positive dendritic cells and strengthen antibody responses compared with non-glycosylated or less optimized antigens [45]. These findings indicate that O-glycans may actively determine how efficiently an antigen enters the antigen-presentation pathway.

Taken together, these observations support a broader view in which O-glycans participate in antigen recognition at multiple levels. They can generate tumor-associated glyco-epitopes, alter the structural presentation of antigenic surfaces, and regulate lectin-mediated uptake by antigen-presenting cells. Through these mechanisms, O-glycosylation helps define whether a molecule is ignored, tolerated, or recognized as immunologically significant. Accordingly, the contribution of O-glycans to immune tolerance should be understood not only in terms of masking or shielding, but also in terms of selective antigen shaping and context-dependent immune visibility [44,45].

### 3.5. Impact of O-Glycans on Pathogen–Host Interactions

O-glycans are central components of the molecular interface between pathogens and host tissues. At mucosal surfaces, they serve not only as structural elements of the glycocalyx and mucus layer, but also as recognition cues that can be exploited or interpreted by viruses, bacteria, and commensal microorganisms. In this context, the contribution of O-glycans to pathogen–host interactions can be understood through three closely connected processes: adhesion, invasion, and immune evasion.

The first step is adhesion. Many microorganisms initiate colonization by recognizing specific glycan motifs on host mucins or other surface glycoproteins. This interaction is not random, but often depends on precise glycan structure, linkage, and local presentation. For example, bacterial lectins can recognize defined fucosylated or sialylated motifs on mucin-type glycans, and such selectivity may determine tissue tropism and colonization efficiency [46,47]. Similarly, microbial binding modules have been shown to preferentially interact with clustered glycan patches on mucins rather than with isolated monosaccharide determinants, indicating that the spatial organization of O-glycans contributes to microbial attachment [48]. These findings suggest that host O-glycosylation patterns help define the initial binding landscape encountered by microbes at epithelial surfaces.

A related principle is seen in the interaction between host mucins and commensal organisms. Akkermansia muciniphila, for instance, exhibits O-glycan-specific binding to mucins, indicating that host glycan composition helps shape microbial localization even in non-pathogenic settings [49]. This is relevant because the same structural features that support commensal colonization may also influence pathogen access, either by competing for binding niches or by altering the biochemical properties of the mucus layer.

Following adhesion, O-glycans may influence pathogen invasion. In some cases, glycans facilitate entry by supporting receptor engagement or by positioning pathogens close to the epithelial surface. In other cases, they function as inhibitory environmental signals. A clear example of the latter is provided by mucus-derived glycans in the intestine, which suppress Salmonella SPI-1-mediated invasion. Rather than serving only as passive decoys, these glycans actively downregulate virulence-associated invasion programs, indicating that host O-glycan-rich environments can shape microbial behavior at the level of gene regulation [50]. Thus, O-glycans do not merely determine whether pathogens bind; they may also influence whether pathogens remain in an adhesive state or proceed to active invasion.

Viral systems provide additional examples of glycan-dependent invasion mechanisms. Viral envelope proteins are frequently O-glycosylated, and these modifications may affect receptor interaction, protein stability, and fusion competence. Site-specific glycoproteomic studies have shown that herpesviruses, including varicella-zoster virus, human cytomegalovirus, and Epstein–Barr virus, carry extensive O-glycosylation on envelope proteins [51]. Likewise, SARS-CoV-2 spike proteins display defined site-specific glycosylation patterns that vary with protein source and viral context [52]. More broadly, analyses of viral envelope proteins support the view that O-linked glycans contribute to the structural and functional properties of viral surface proteins, particularly in regions involved in host interaction or immune exposure [53]. These data indicate that O-glycosylation is not a peripheral modification in viruses, but part of the molecular architecture that governs host entry and interface stability.

The third major dimension is immune evasion. Because O-glycans can cover exposed peptide regions or alter surface topology, they may shield viral or microbial antigens from host immune recognition. This is especially relevant for heavily glycosylated envelope or mucin-like domains. Experimental evidence from respiratory syncytial virus has shown that limiting O-glycosylation within a mucin-like domain of the G glycoprotein enhances the immunogenicity and protective efficacy of the antigen, implying that dense O-glycosylation normally reduces immune accessibility [54]. Similarly, identification of novel O-linked glycans on tick-borne encephalitis virus envelope proteins further supports the notion that complex viral O-glycosylation may influence antigen presentation and host recognition [55].

Host glycosylation can also affect immune control of infection from the opposite direction. Glycoengineered keratinocyte models have shown that specific host glycans are required during multiple stages of HSV-1 infection, demonstrating that host glycan composition can determine viral permissiveness as well as subsequent infectious progression [56]. In this sense, O-glycans participate in pathogen–host interactions in a bidirectional manner: pathogens exploit host glycans for adhesion and entry, while host glycan organization can either restrict invasion or modify immune detectability.

Taken together, O-glycans influence pathogen–host interactions through a coordinated sequence of effects on adhesion, invasion, and immune evasion. They provide microbial binding platforms, regulate the transition from colonization to invasion, and shape antigen exposure at the host–pathogen interface. This makes O-glycosylation a central determinant of how infection begins and how it is subsequently interpreted by the immune system.

## 4. O-Glycosylation in Disease

### 4.1. O-Glycosylation in Cancer

Aberrant O-glycosylation is a characteristic feature of malignant transformation and influences tumor progression at multiple levels. In cancer, its effects are not limited to surface glycan remodeling, but extend to receptor signaling, cell adhesion, metabolic adaptation, immune escape, and therapeutic responsiveness. Based on the evidence presented in the current literature, the role of O-glycosylation in cancer can be organized into three major aspects: truncation of mucin-type O-glycans, dysregulation of the O-GlcNAc metabolic axis, and the pathogenic as well as translational relevance of mucin family glycoproteins.

One of the best-recognized cancer-associated glycosylation changes is the accumulation of truncated O-glycan structures, especially Tn and sTn antigens. These glycans arise when normal elongation of mucin-type O-glycans is disrupted, resulting in exposure of immature glycan motifs on the tumor-cell surface. Such truncation is not only a diagnostic hallmark, but also functionally relevant to tumor behavior. In breast cancer, Tn antigen is enriched in metastatic lesions and is associated with lymph node metastasis and poor prognosis. Experimental induction of Tn expression through Cosmc disruption promotes migration and invasion, whereas targeting Tn antigen suppresses abnormal O-glycosylation-driven metastasis [57]. These observations support the view that truncated O-glycans can actively contribute to epithelial plasticity, altered adhesion, and invasive dissemination rather than merely reflecting malignant status.

Abnormal O-glycosylation also affects tumor progression through altered glycosyltransferase activity. C1GALT1, a key enzyme for core 1 O-glycan synthesis, is highly expressed in pancreatic ductal adenocarcinoma and correlates with poor patient survival. Mechanistically, elevated C1GALT1 enhances integrin β1 O-glycosylation and promotes invasive behavior, indicating that altered glycan elongation can modify tumor–cell interaction with the extracellular matrix and increase motility [9]. Likewise, GALNT8 has been shown to suppress breast-cancer metastatic potential through modulation of EGFR O-GalNAcylation, suggesting that site-specific O-glycosylation of growth factor receptors may shape downstream signaling outputs relevant to tumor dissemination [58]. Together, these findings indicate that changes in O-glycosyltransferase expression are not merely correlative, but may redirect receptor function and adhesion programs in ways that favor or restrain metastasis depending on context.

A second major aspect of cancer-associated glycosylation is dysregulation of O-GlcNAcylation. Unlike mucin-type O-glycosylation in the secretory pathway, O-GlcNAcylation occurs dynamically in the cytoplasm and nucleus and is tightly coupled to nutrient availability through the hexosamine biosynthetic pathway. In cancer cells, this modification is frequently elevated and supports metabolic adaptation, stress tolerance, and survival signaling. In hepatocellular carcinoma, enhanced O-GlcNAcylation promotes malignant progression and is linked to increased proliferative and survival capacity [59]. More importantly, recent studies indicate that O-GlcNAcylation also modulates how tumor cells respond to therapy-induced stress and programmed cell death.

This is particularly evident in the context of ferroptosis and chemotherapy response. In glioblastoma, pharmacological inhibition of the hexosamine biosynthetic pathway with FR054 increases sensitivity to temozolomide by reducing O-GlcNAcylation and promoting ferroptosis-related changes [60]. A related study showed that inhibition of O-GlcNAcylation decreases cell viability and autophagy and enhances temozolomide sensitivity in glioblastoma cells [61]. In colorectal cancer, suppression of O-GlcNAcylation shifts the cellular response to chemotherapy from senescence toward apoptosis, indicating that this modification can determine how cancer cells allocate stress responses under treatment [62]. These results suggest that O-GlcNAcylation functions as a regulator of therapy-induced cell-fate decisions rather than simply a marker of tumor metabolism.

The relationship between O-GlcNAcylation and ferroptosis is particularly instructive because it highlights the context dependence of this modification. In hepatocellular carcinoma, O-GlcNAcylation stabilizes the transferrin receptor TFRC and thereby affects iron metabolism and ferroptotic sensitivity [63]. In pancreatic cancer, O-GlcNAcylation and deubiquitination cooperate to stabilize METTL3, promote HMGB1 degradation, suppress ferroptosis, and enhance gemcitabine resistance [64]. These findings illustrate that the oncogenic effect of O-GlcNAcylation is not uniform, but depends on the specific substrate and signaling context involved.

#### Therapeutic Targeting of Mucin-Associated O-Glycosylation in Cancer

A third important aspect of cancer-associated O-glycosylation is the translational relevance of mucin family glycoproteins. MUC1, MUC4, and MUC16 are heavily O-glycosylated, frequently overexpressed or structurally altered in tumors, and contribute to the formation of an abnormal tumor-cell surface. Their large extracellular domains and dense glycan coverage can influence receptor accessibility, immune recognition, cell–cell interaction, and the tumor microenvironment. These features make mucin-associated O-glycosylation not only a mechanism of tumor progression, but also a potential source of diagnostic and therapeutic targets.

MUC1-C is one of the most actively explored mucin-related therapeutic targets. Recent evidence shows that MUC1-C-directed antibody–drug conjugates can suppress KRAS inhibitor-resistant pancreatic cancer, suggesting that mucin-associated molecules may remain therapeutically accessible even when classical oncogenic pathways become drug resistant [65]. MUC4 has also shown translational potential, particularly in tumor imaging. Near-infrared dye-labeled anti-MUC4 antibodies can clearly target pancreatic cancer liver metastases and peritoneal lesions, supporting the value of MUC4 as an imaging-associated surface marker for metastatic pancreatic cancer [66].

MUC16 represents another clinically relevant mucin target, especially for cellular immunotherapy. CAR-T cells targeting the CA125 extracellular repeat domain of MUC16 can recognize and kill MUC16-positive tumor cells [67]. However, antigen heterogeneity remains a major obstacle in solid tumors. To address this problem, tandem CAR-T cells targeting both mesothelin and MUC16 have been developed to improve coverage of heterogeneous tumor-cell populations [68]. This strategy illustrates how mucin-targeted therapy may need to move beyond single-antigen recognition toward more flexible antigen-combination approaches.

Despite these promising advances, several challenges remain. Mucin expression is often heterogeneous across tumors and metastatic sites, which may lead to incomplete targeting or immune escape. On-target/off-tumor toxicity is also a concern because some mucins are expressed in normal epithelial tissues. In addition, therapeutic specificity may depend not only on the mucin protein backbone, but also on tumor-associated glycoforms or composite glycopeptide epitopes. The dense glycocalyx and immunosuppressive tumor microenvironment may further restrict antibody penetration or immune-cell activity. Therefore, future mucin-targeted strategies will require careful patient stratification based on tumor type, antigen abundance, glycoform state, and lesion distribution.

Overall, MUC1, MUC4, and MUC16 illustrate three complementary translational directions for mucin-associated O-glycosylation: antibody–drug conjugate-based therapy, imaging-guided tumor detection, and CAR-T-cell immunotherapy. These examples support the view that aberrant mucin-associated O-glycosylation is not only involved in tumor progression, but may also provide clinically actionable targets.

**Figure 2:**
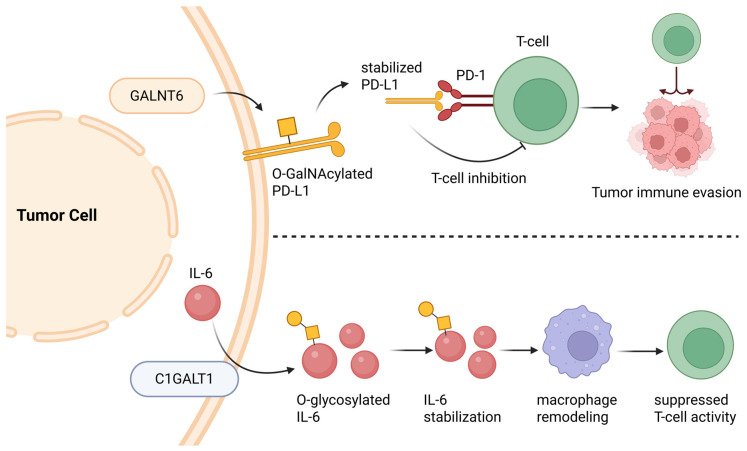
Mucin-type O-GalNAc glycosylation promotes tumor immune evasion through PD-L1 stabilization and IL-6-dependent immune remodeling. This schematic illustrates two representative mechanisms by which tumor-associated mucin-type O-GalNAc glycosylation contributes to immune escape. In the upper pathway, GALNT6 mediates O-GalNAc glycosylation of PD-L1 on tumor cells. This modification stabilizes PD-L1, enhances PD-1/PD-L1 checkpoint engagement with T cells, and suppresses T-cell activity, thereby promoting tumor immune evasion. In the lower pathway, C1GALT1-dependent core 1 O-GalNAc glycosylation stabilizes IL-6 and sustains IL-6-mediated immune remodeling. Stabilized IL-6 can promote macrophage remodeling and contribute to an immunosuppressive tumor microenvironment, leading to reduced T-cell activity. Together, these pathways show that aberrant mucin-type O-GalNAc glycosylation can regulate tumor immunity both by directly stabilizing immune checkpoint proteins and by reshaping cytokine-dependent tumor–immune cell communication. Abbreviations: C1GALT1, core 1 β1,3-galactosyltransferase 1; GALNT6, polypeptide N-acetylgalactosaminyltransferase 6; IL-6, interleukin-6; O-GalNAc, O-linked N-acetylgalactosamine; PD-1, programmed cell death protein 1; PD-L1, programmed death-ligand 1.

Finally, aberrant O-glycosylation also contributes to tumor immune evasion. As discussed in earlier sections, O-glycosylation can stabilize PD-L1 and strengthen immunosuppressive signaling, while altered glycosylation-dependent cytokine networks can remodel macrophage and T-cell behavior in the tumor microenvironment [36,37]. Together, these findings indicate that tumor-associated mucin-type O-GalNAc glycosylation can promote immune evasion through both checkpoint-protein stabilization and cytokine-dependent immune remodeling (Figure 2).

### 4.2. O-Glycosylation in Autoimmune Diseases

Aberrant O-glycosylation contributes to autoimmune disease through at least two related mechanisms. First, it can alter the structure and immune behavior of circulating molecules, especially immunoglobulins, thereby promoting pathogenic immune complex formation or abnormal receptor interaction. Second, it can affect immune-cell tolerance, trafficking, and tissue homeostasis by reshaping glycan-dependent signaling at the cellular level. Among autoimmune disorders, IgA nephropathy provides the clearest and best-characterized example of how defective O-glycosylation is translated into disease.

#### 4.2.1. IgA Nephropathy

IgA nephropathy is closely linked to abnormal O-glycosylation of IgA1. The defining molecular abnormality is the generation of galactose-deficient O-glycans in the hinge region of IgA1, which alters the biochemical properties of the molecule and promotes recognition by antiglycan antibodies. This leads to the formation of circulating immune complexes that are prone to mesangial deposition, complement activation, and glomerular injury [69]. In this disease, aberrant O-glycosylation is therefore not a secondary marker of inflammation, but a central pathogenic event.

The development of this abnormal IgA1 glycoform is associated with defects in the enzymatic machinery that controls O-glycan biosynthesis. Altered activity of GalNAc transferases and glycan-processing enzymes has been implicated in the incomplete maturation of IgA1 O-glycans. In particular, GALNT14 deficiency has been proposed to connect several steps in the pathogenic chain of IgA nephropathy, further supporting the idea that dysregulated O-glycosylation is mechanistically involved in disease initiation and progression rather than merely accompanying established renal injury [70].

Importantly, the relevance of O-glycosylation in IgA nephropathy is not confined to the IgA1 molecule itself. Defects in O-glycosylation may also influence the immune architecture that supports abnormal IgA responses. Loss of GalNAc-T14 has been linked to altered B-cell homing in the context of IgA nephropathy, suggesting that glycosylation-dependent changes in immune-cell trafficking may contribute to the maintenance of pathogenic IgA production [42]. This is important because it broadens the disease model from an antibody-centered mechanism to a more integrated view in which glycosylation defects affect both humoral products and the immune compartments that generate them.

Further support for the pathogenic importance of IgA O-glycosylation comes from related IgA-mediated disorders. Mass spectrometric analysis in Henoch–Schönlein purpura nephritis has revealed altered IgA1 O-glycosylation patterns, indicating that abnormal hinge-region glycosylation is not unique to classical IgA nephropathy but may be part of a broader spectrum of IgA-associated immune dysregulation [71]. Together, these findings reinforce the view that O-glycosylation defects are functionally important determinants of pathogenic IgA biology.

Taken together, IgA nephropathy represents the most direct example in which defective mucin-type O-glycosylation can be linked to a well-defined autoimmune disease mechanism. Abnormal IgA1 glycoforms, antiglycan immune recognition, immune complex deposition, and glomerular inflammation form a coherent pathogenic sequence that places O-glycosylation at the center of disease development [69,70].

#### 4.2.2. Other Autoimmune and Inflammatory Disorders

Although IgA nephropathy is the most established example, altered O-glycosylation is also implicated in other autoimmune and chronic inflammatory conditions. In Sjögren’s syndrome, changes in salivary mucin O-glycans have been detected, suggesting that abnormal glycosylation may contribute to impaired mucosal lubrication and barrier dysfunction [72]. In parallel, altered Golgi localization of Gal3-O-sulfotransferases affects mucin sulfation in salivary glands from patients with Sjögren’s syndrome, indicating that defective glycan processing may participate in exocrine dysfunction at the tissue level [73].

At the cellular immune level, disruption of O-glycan biosynthesis can also weaken tolerance. Cosmc deficiency impairs normal core 1 O-glycan synthesis and has been shown to cause spontaneous autoimmunity by disrupting B-cell tolerance [43]. This finding is important because it demonstrates that abnormal glycosylation can act upstream of overt disease by changing the activation threshold of immune cells themselves. Consistent with this broader view, aberrant B-cell glycosylation has been proposed as a potential pathogenic factor and therapeutic entry point in autoimmunity more generally [74].

O-glycosylation-related mechanisms may also contribute to chronic inflammatory joint disease. In synovial fibroblasts, aberrant relocation of GalNAc transferase activity to the endoplasmic reticulum drives pathological O-glycosylation and promotes cartilage degradation, providing an example in which altered glycosylation contributes to tissue destruction in inflammatory disease [75]. In osteoarthritis, altered UDP-GlcNAc handling and O-GlcNAcylation regulate senescence-associated secretory phenotypes, linking glycosylation-related metabolic control to chronic joint inflammation [76]. Although these disorders are not classic antibody-mediated autoimmune diseases in the same sense as IgA nephropathy, they support the broader conclusion that glycosylation abnormalities can participate in immune-driven tissue pathology across multiple disease settings.

Overall, the evidence indicates that O-glycosylation contributes to autoimmune disease not through a single universal mechanism, but through context-dependent effects on antibodies, immune-cell tolerance, tissue barriers, and inflammatory remodeling. Among these conditions, IgA nephropathy remains the clearest model of direct pathogenic O-glycosylation, while other autoimmune and inflammatory diseases point to a wider role of glycan dysregulation in immune-mediated pathology.

**Figure 3:**
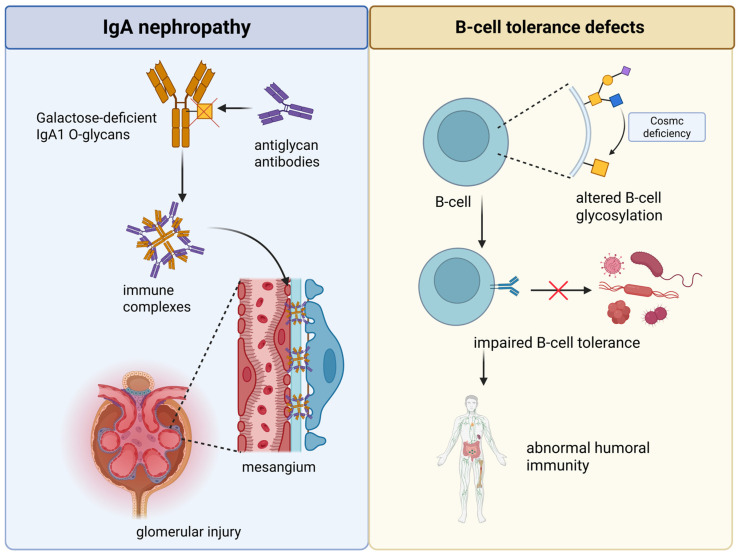
Aberrant mucin-type O-GalNAc glycosylation disrupts humoral immune homeostasis and promotes autoimmune pathology. This schematic illustrates two representative autoimmune-related mechanisms involving defective mucin-type O-GalNAc glycosylation. In IgA nephropathy, abnormal hinge-region O-GalNAc glycans on IgA1 are characterized by reduced galactosylation, generating galactose-deficient IgA1. These aberrant IgA1 O-glycans can be recognized by antiglycan antibodies, leading to immune-complex formation, mesangial deposition, and glomerular injury. In parallel, Cosmc deficiency impairs core 1 O-glycan biosynthesis and alters B-cell glycosylation. This defective glycosylation state disrupts B-cell tolerance and contributes to abnormal humoral immunity. Together, these mechanisms show that mucin-type O-GalNAc glycosylation is important for maintaining antibody homeostasis, B-cell tolerance, and tissue-specific immune balance. Arrows indicate the progression of pathogenic processes; dashed lines indicate indirect or defective regulatory links; red cross indicate reduced or impaired glycan extension. Abbreviations: Cosmc, core 1 β1,3-galactosyltransferase-specific molecular chaperone; IgA1, immunoglobulin A1; O-GalNAc, O-linked N-acetylgalactosamine.

The roles of aberrant IgA1 O-glycosylation and Cosmc-dependent B-cell glycosylation defects in autoimmune pathology are summarized in Figure 3.

### 4.3. O-Glycosylation and Infectious Diseases

O-glycosylation plays a broad role in infectious diseases by influencing how pathogens attach to host tissues, how mucosal barriers are maintained or disrupted, and how host immune systems recognize and clear infection. At mucosal surfaces in particular, O-glycans form a major part of the molecular environment encountered by microbes. They are therefore positioned at the interface between host defense and pathogen adaptation. In this context, the contribution of O-glycosylation to infectious disease can be organized into three closely related aspects: pathogen adhesion and invasion, alteration of mucosal barrier function, and regulation of immune recognition and clearance.

#### 4.3.1. Pathogen Adhesion and Invasion

The earliest stage of many infections depends on successful attachment to host tissues, and O-glycans often participate directly in this process. Mucin-type O-glycans on epithelial surfaces and in mucus provide recognition motifs for viruses, bacteria, and commensal organisms. These interactions are not determined simply by the presence of glycans, but by their precise structure, terminal modification, and local presentation.

Several studies support this principle. Bacterial lectins can recognize defined motifs carried by mucin-type glycans, including fucosylated glycopeptide structures, indicating that O-glycan fine structure helps determine microbial binding specificity [46]. Likewise, work on bacterial adhesins has shown that recognition of eukaryotic glycans can be highly selective and structurally constrained, as illustrated by Mycoplasma-related adhesion systems [47]. More broadly, microbial binding modules can preferentially attach to clustered glycan patches on mucins, showing that spatial glycan organization contributes to colonization efficiency [48].

Host–microbe interactions at this level are not restricted to pathogens. Akkermansia muciniphila exhibits O-glycan-specific binding to mucins, illustrating that host glycan composition shapes microbial localization even under commensal conditions [49]. This is relevant to infectious disease because the glycan structures that help organize normal microbial ecology may also influence susceptibility to pathogen attachment.

After adhesion, O-glycans may affect whether invasion proceeds. In some settings, mucus-derived glycans act not as passive decoys but as active regulators of pathogen virulence. A representative example is provided by Salmonella, in which mucus-derived glycans suppress SPI-1-mediated invasion, demonstrating that host glycans can directly inhibit the transition from colonization to epithelial penetration [50]. Thus, O-glycans can influence infection not only by enabling microbial contact, but also by determining whether invasive programs are activated.

Viruses provide an additional layer of complexity because their own surface proteins may be O-glycosylated. Global mapping of herpesvirus envelope protein glycosylation has shown extensive O-glycan modification in varicella-zoster virus, human cytomegalovirus, and Epstein–Barr virus [51]. SARS-CoV-2 spike proteins also display site-specific glycosylation patterns that differ according to viral context and protein source [52]. More generally, the structural and functional importance of O-linked glycans on viral envelope proteins has been recognized as a recurring feature relevant to receptor interaction, protein conformation, and host entry [53]. Together, these findings indicate that O-glycosylation participates in both sides of the host–pathogen interface: host glycans can determine microbial binding, and pathogen glycans can modulate the molecular machinery of entry.

#### 4.3.2. Altered Mucosal Barrier Function

Because mucosal surfaces are enriched in heavily O-glycosylated mucins, changes in mucin glycosylation can strongly affect barrier integrity. O-glycans contribute to mucus hydration, viscoelasticity, protease resistance, microbial stratification, and the separation of microorganisms from epithelial cells. Disruption of these structures can therefore alter susceptibility to infection as well as inflammatory injury.

This is particularly well illustrated in the intestine. Reviews of intestinal mucin glycosylation emphasize that glycan composition is a key determinant of mucus structure and mucosal homeostasis [77]. Experimental studies further show that inflammatory cytokines can regulate this process in functionally meaningful ways. IL-22 promotes mucin-type O-glycosylation and supports intestinal recovery, indicating that enhancement of mucin glycosylation is part of the protective mucosal response to injury [78]. Similarly, B3GNT7 has been shown to regulate mucin O-glycosylation and alleviate colonic inflammation, supporting the view that glycan elongation pathways contribute directly to barrier preservation [79].

These findings are important in the context of infectious disease because impaired mucus organization can influence both pathogen access and persistence. A weakened or compositionally altered O-glycan barrier may expose epithelial surfaces to microbial contact more readily, whereas restoration of mucin glycosylation can reinforce mucosal separation and reduce pathological invasion. In this sense, O-glycosylation is not simply part of epithelial maintenance; it is an active determinant of resistance at the frontline of infection.

#### 4.3.3. Immune Recognition and Clearance

Beyond adhesion and barrier function, O-glycosylation also influences how infection is sensed and controlled by the immune system. This occurs at least in part through O-GlcNAcylation-dependent regulation of innate immune signaling pathways. Compared with the barrier-related roles of mucin-type O-glycosylation, these mechanisms operate more at the level of intracellular immune signaling and can shape whether host defense is amplified or restrained.

A key example is STING, an essential mediator of antiviral innate immunity. O-GlcNAc modification of STING has been shown to promote its signaling activity and support antiviral responses, demonstrating that glycosylation contributes directly to the machinery of pathogen sensing [80]. More broadly, O-GlcNAcylation has been implicated in the regulation of innate immune-cell function, suggesting that glycosylation may tune inflammatory and antiviral signaling in macrophages and related cell types [81].

**Figure 4:**
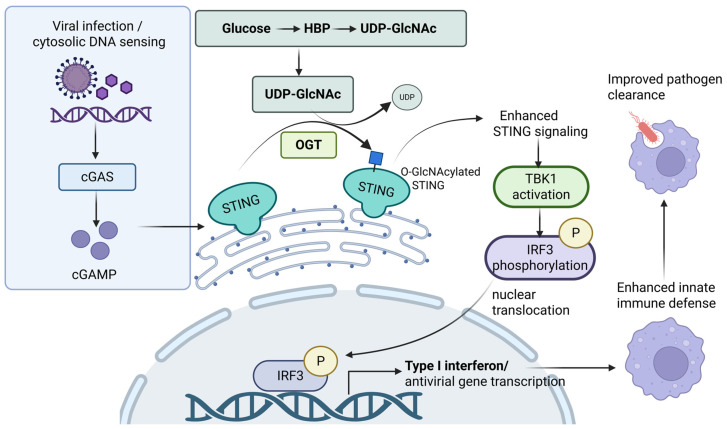
O-GlcNAcylation of STING enhances antiviral innate immune signaling and promotes pathogen clearance. Viral infection or cytosolic DNA activates the cGAS–cGAMP–STING pathway at the endoplasmic reticulum-associated cytosolic interface. In parallel, glucose metabolism through the hexosamine biosynthetic pathway (HBP) generates UDP-GlcNAc, which serves as the donor substrate for O-GlcNAc transferase (OGT). OGT adds a single GlcNAc residue to STING, generating O-GlcNAcylated STING. This intracellular monosaccharide modification enhances STING signaling, promotes TBK1 activation and IRF3 phosphorylation, and facilitates IRF3 nuclear translocation. Activated IRF3 then drives type I interferon and antiviral gene transcription, thereby strengthening innate immune defense and improving pathogen clearance. This pathway highlights that O-GlcNAcylation is distinct from mucin-type O-GalNAc glycosylation: it is a reversible intracellular modification involving a single GlcNAc residue rather than an extended extracellular glycan chain. Arrows indicate the direction of pathway activation, signaling flow, nuclear translocation, and functional immune outcomes in the cGAS–cGAMP–STING–TBK1–IRF3 axis. Abbreviations: cGAMP, cyclic GMP–AMP; cGAS, cyclic GMP–AMP synthase; GlcNAc, N-acetylglucosamine; HBP, hexosamine biosynthetic pathway; IRF3, interferon regulatory factor 3; OGT, O-GlcNAc transferase; STING, stimulator of interferon genes; TBK1, TANK-binding kinase 1; UDP-GlcNAc, uridine diphosphate N-acetylglucosamine.

In host cells, O-GlcNAcylation of STING strengthens downstream TBK1/IRF3 signaling, leading to increased antiviral gene transcription. The resulting enhancement of innate immune defense promotes more effective pathogen clearance. This pathway highlights a mechanistic role for O-GlcNAcylation in linking intracellular glycosylation-dependent signaling to host defense against infection. The role of STING O-GlcNAcylation in antiviral innate immune signaling is summarized in Figure 4.

Host glycosylation can also influence infection susceptibility at the cellular interface. Glycoengineered keratinocyte libraries have shown that specific glycan structures are required at multiple stages of HSV-1 infection, indicating that host glycan composition affects not only pathogen binding but also downstream progression of infection [56]. In this sense, O-glycosylation contributes to immune clearance indirectly as well, because host cells with different glycan landscapes may differ in permissiveness, immune detectability, and infection outcome.

Taken together, these observations indicate that O-glycosylation shapes infectious disease through a coordinated set of mechanisms. It affects whether pathogens bind and invade, whether mucosal barriers remain protective, and whether host immune systems mount effective recognition and clearance. Rather than acting at a single stage of infection, O-glycans influence the entire progression from host encounter to immune control.

### 4.4. Genotype–Phenotype Correlations in Glycosylation-Related Disorders

Genetic defects in glycosylation-related enzymes and processing factors provide direct evidence that altered glycan biosynthesis can produce clinically meaningful phenotypes. Although this review focuses mainly on O-linked glycosylation-related immune regulation, selected N-glycosylation and O-linked glycosylation disorders are included here to illustrate the broader genotype–phenotype framework of glycosylation defects. These examples show that pathogenic variants may impair glycan initiation, elongation, processing, or signaling-dependent glycoprotein function, leading to tissue-specific or multisystem disease manifestations (Table 1).

## 5. Emerging Technologies for O-Glycan and O-GlcNAcylation Analysis

### 5.1. Analytical Distinction Between Mucin-Type O-Glycosylation and O-GlcNAcylation

A clear methodological distinction between mucin-type O-glycosylation and O-GlcNAcylation is essential for interpreting glycosylation studies. Although both modifications occur on serine or threonine residues, they differ in cellular compartment, glycan structure, enzymatic regulation, and analytical requirements. Mucin-type O-GalNAc glycosylation is initiated mainly in the Golgi apparatus and can be extended into diverse O-glycan chains. Therefore, its analysis requires simultaneous characterization of the peptide backbone, glycosylation site, site occupancy, and attached glycan structures.

By contrast, O-GlcNAcylation is a dynamic intracellular modification in which a single GlcNAc residue is added to nuclear, cytoplasmic, or mitochondrial proteins by OGT and removed by OGA. It does not generate elongated glycan chains and is functionally closer to a reversible signaling modification than to secretory-pathway glycan maturation. Thus, methods used for extended mucin-type O-glycans cannot be directly applied to O-GlcNAcylation without adjustment. For mucin-type O-glycosylation, the central questions are which protein is modified, which site carries the glycan, and which glycoform is present. For O-GlcNAcylation, the main questions are which intracellular proteins are modified, how modification levels change dynamically, and whether OGT/OGA-dependent cycling alters signaling output. The major analytical strategies for mucin-type O-GalNAc glycosylation and O-GlcNAcylation, together with their main applications and limitations, are summarized below (Table 2).

### 5.2. LC–MS/MS-Based Strategies for O-Glycan and O-Glycopeptide Analysis

LC–MS/MS has become a central platform for O-glycan and O-glycopeptide analysis because it can combine chromatographic separation, mass measurement, fragmentation-based structural inference, and site localization. For mucin-type O-glycosylation, this approach is particularly valuable because it can retain information from both the peptide backbone and the attached glycan, allowing researchers to distinguish altered protein abundance from altered glycosylation occupancy or glycoform composition [82].

Different LC strategies provide complementary advantages. Reversed-phase LC is widely used for glycopeptide analysis because it separates species mainly according to peptide properties and is compatible with complex biological samples. HILIC can enrich or separate hydrophilic glycopeptides and released glycans, improving detection of low-abundance glycosylated species. Porous graphitized carbon LC is useful for released glycan analysis and can improve separation of isomeric structures, although it is technically more demanding. Therefore, the choice of LC strategy should depend on whether the main goal is site localization, glycoform profiling, or structural discrimination.

Fragmentation strategy is equally important. CID and HCD efficiently generate glycan-derived diagnostic ions and are useful for identifying glycopeptides, but they may preferentially fragment glycosidic bonds and provide insufficient peptide backbone information for site localization. ETD and EThcD are more useful for localizing O-glycosylation sites because they better preserve labile glycan modifications while fragmenting the peptide backbone. UVPD can provide rich structural information and is especially useful for complex glycopeptides, although it remains less widely available than HCD or EThcD [82,89].

The major strength of LC–MS/MS-based analysis is that it connects glycan composition with peptide sequence and, in favorable cases, modification site. However, several limitations remain. Glycan lability can reduce localization confidence, isomeric glycoforms are difficult to distinguish, mucin-rich domains are often poorly covered, and site-specific quantification is still challenging. Therefore, LC–MS/MS results should be interpreted with attention to fragmentation mode, enrichment strategy, protein abundance, and validation method.

### 5.3. Complementary Workflows for Site Localization and Structural Resolution

Several workflows have been developed to improve O-glycoproteomic analysis. Bottom-up glycoproteomics remains the most widely used approach. Proteins are digested into peptides or glycopeptides before MS analysis, enabling broad sample compatibility and relatively high throughput. This strategy is useful for identifying O-glycosylated proteins and mapping candidate glycosylation sites, but it may lose information about how multiple O-glycans are organized across larger mucin-like domains [82].

Middle-down strategies preserve longer glycopeptide fragments and therefore provide better regional context. They are particularly useful for heavily glycosylated or repetitive domains where neighboring glycosylation events may function together. However, larger glycopeptides are more difficult to separate, fragment, and annotate. Top-down analysis preserves intact glycoprotein information and can theoretically reveal complete proteoform architecture, but it remains technically demanding for large and heterogeneous glycoproteins.

Recent innovations have partly addressed these limitations. O-glycoproteases can improve access to densely glycosylated regions that are poorly represented in conventional tryptic workflows, especially mucin-rich sequences [83]. Ion mobility adds an additional separation dimension and can improve discrimination of isomeric or near-isomeric O-glycans [90]. Bioinformatic tools have also improved glycopeptide annotation, site-localization scoring, and integration of peptide and glycan evidence [89]. Together, these methods have expanded the ability to analyze O-glycosylation beyond simple protein identification toward more site- and structure-aware interpretation.

### 5.4. Detection and Functional Validation of O-GlcNAcylation

O-GlcNAcylation requires a distinct analytical framework because it involves a single intracellular GlcNAc residue rather than an extended glycan chain. Antibody-based detection is commonly used for global or protein-specific O-GlcNAc analysis by immunoblotting, immunoprecipitation, or imaging. However, antibody approaches may show substrate bias and usually do not provide site-level information.

Chemoenzymatic labeling provides a more selective strategy. In these workflows, engineered enzymes introduce a chemical handle onto O-GlcNAc residues, allowing enrichment, visualization, or MS-based identification. When combined with LC–MS/MS, this approach can support site-specific analysis, although confident localization still depends on peptide sequence and fragmentation quality. ETD and EThcD are especially useful because they help preserve the labile O-GlcNAc modification while generating peptide backbone fragments.

Functional validation is essential in O-GlcNAcylation studies. Because OGT and OGA regulate many substrates, global changes in O-GlcNAcylation do not automatically identify the responsible protein or pathway. Pharmacological inhibition, genetic knockdown, overexpression, or targeted manipulation of OGT/OGA can help test whether a phenotype depends on O-GlcNAc cycling, but broad perturbation may cause indirect effects. Therefore, stronger studies combine detection, site mapping, dynamic profiling, and substrate-specific functional validation.

Recent developments have improved this area. Spatiotemporal activation strategies allow more precise manipulation of O-GlcNAcylation in living cells, while spatial and temporal proteomics reveal distinct distributions and dynamics of O-GlcNAcylated proteins [84,85]. In addition, studies on OGT/OGA substrate selection and targeted protein O-GlcNAcylation suggest that future work may move from global perturbation toward substrate-specific interrogation [86,87,91].

In addition to analytical detection, functional glycoengineering approaches provide an important complementary strategy for validating glycosylation-dependent immune mechanisms. Lectin-based assays, glycan-binding protein probes, and antibody-based detection can reveal whether specific glycan motifs are accessible for immune recognition, although these methods usually lack the site-level resolution of LC–MS/MS. Metabolic oligosaccharide engineering, chemoenzymatic cell-surface labeling, and glycan-remodeling strategies can further be used to alter glycan display, linkage, or density and then test the resulting effects on lectin binding, immune-cell activation, or receptor signaling. These approaches are particularly valuable for glycoimmunology because many immune effects depend not only on the presence of a glycan structure, but also on its spatial presentation, clustering, and multivalent interaction with glycan-binding proteins. However, such perturbation strategies should be interpreted carefully because global glycan remodeling may affect multiple pathways simultaneously.

### 5.5. Current Limitations and Future Technical Needs

Despite major progress, important limitations remain. For mucin-type O-glycosylation, site-specific quantification is still difficult, especially in low-abundance or highly heterogeneous glycopeptides. Isomer discrimination also remains incomplete, even when ion mobility or specialized LC strategies are used. Dense mucin-like domains are particularly challenging because clustered O-glycans reduce protease accessibility and complicate spectral interpretation.

For O-GlcNAcylation, the major limitation is functional specificity. Global OGT or OGA perturbation affects many proteins and may generate secondary effects. Therefore, future studies need improved substrate-specific tools, better site-localization confidence, and stronger integration between MS-based identification and biological validation.

Another shared problem is standardization. Different enrichment strategies, fragmentation methods, scoring systems, and reporting criteria can produce datasets that are difficult to compare across laboratories. This is especially important for translational research, where reproducibility is essential.

Future technical development should therefore focus on three priorities: improving site-resolved quantification, resolving glycan structural ambiguity, and linking glycosylation changes to defined biological functions. Importantly, mucin-type O-glycosylation and O-GlcNAcylation should be analyzed with distinct but complementary methodological frameworks. Only by preserving this distinction can glycosylation studies move from descriptive profiling toward mechanistic and clinically interpretable conclusions.

## 6. Challenges and Future Directions

### 6.1. Limited Spatiotemporal Resolution of O-Glycosylation Dynamics

A major challenge in current O-glycosylation research is that most available data remain largely static, whereas O-glycosylation itself is often highly dynamic and context dependent. This problem is especially evident for O-GlcNAcylation, which can respond rapidly to nutrient status, stress, signaling input, and subcellular redistribution. As a result, many studies can identify whether a protein is glycosylated under a given condition, but cannot fully resolve when the modification occurs, where it occurs within the cell, or how quickly it changes during signaling or disease progression.

This limitation has important consequences for mechanistic interpretation. Without sufficient temporal and spatial resolution, it is difficult to distinguish causal regulatory events from downstream or compensatory changes. In many cases, glycosylation differences are measured at an endpoint, after substantial remodeling has already occurred. Such data are valuable for association, but they do not necessarily reveal whether a given O-glycosylation event initiates a biological response, stabilizes an ongoing state, or simply reflects broader cellular reprogramming. This issue is particularly relevant in immune signaling and tumor biology, where signaling pathways can shift rapidly and where the functional meaning of a modification may depend strongly on timing and localization.

Recent methodological work has begun to address this problem, but the field has not yet fully overcome it. Optogenetic or chemically controlled strategies for activating O-GlcNAcylation in living cells have demonstrated that O-GlcNAc dynamics can be manipulated with improved temporal precision, providing a way to move beyond endpoint measurements [84]. In parallel, spatiotemporal proteomic analyses have shown that O-GlcNAcylated proteins are not uniformly distributed, but instead display distinct localization patterns and dynamic behavior across subcellular compartments [85]. These studies are important because they indicate that O-glycosylation is not only condition-specific but also compartment-specific, and that its biological effects cannot always be inferred from bulk measurements alone.

Even so, current approaches still have clear limitations. Most high-resolution methods remain technically specialized, are difficult to scale to complex tissues, and are more developed for O-GlcNAcylation than for mucin-type O-glycosylation in the secretory pathway. This means that dynamic mapping is still much less mature for many extracellular or membrane-associated O-glycoproteins, even though these molecules are central to receptor regulation, immune recognition, and host–pathogen interaction. In heavily glycosylated regions such as mucin domains, it remains especially difficult to determine how site occupancy and glycoform composition change over time in a physiologically meaningful manner.

Future progress will depend on integrating temporal control, spatial resolution, and site-resolved glycoproteomics more effectively. In practical terms, this means combining perturbation tools with high-resolution analytical workflows so that glycosylation changes can be linked directly to defined biological transitions rather than inferred retrospectively. More broadly, the field needs approaches that can capture O-glycosylation as a dynamic regulatory process rather than as a static molecular feature. Until that becomes routine, limited spatiotemporal resolution will remain a major barrier to assigning precise function to many O-glycosylation events [84,85].

### 6.2. Incomplete Understanding of Enzyme Substrate Selection

A second major challenge in the field is that substrate selection by O-glycosylation-related enzymes remains incompletely understood. This problem affects both mucin-type O-glycosylation in the secretory pathway and O-GlcNAcylation in the cytoplasm and nucleus. Although numerous glycosylated proteins and modification sites have now been identified, it is still often unclear why a given protein is modified in one cellular context but not in another, or why apparently similar substrates show different glycosylation patterns. As a result, the predictive interpretation of O-glycosylation remains limited.

For mucin-type O-glycosylation, this uncertainty is partly due to the lack of a simple sequence rule. Initiation of O-GalNAc addition depends on the ppGalNAc-transferase family, but modification cannot be predicted reliably from primary amino acid sequence alone. Local protein structure, neighboring residues, enzyme isoform distribution, Golgi organization, and competition among nearby sites may all influence site occupancy. This complexity makes it difficult to define clear substrate-selection principles and helps explain why proteins with similar serine/threonine-rich regions may still exhibit markedly different glycosylation patterns. Structural work on GalNAc recognition further supports the idea that molecular recognition is shaped by more than a short peptide motif, emphasizing the importance of local structural context in determining whether glycosylation occurs [92].

A related issue exists for O-GlcNAcylation. Although OGT and OGA are the central cycling enzymes, their functional selectivity cannot be explained simply by the presence of serine or threonine residues. Current evidence indicates that substrate recognition is influenced by a combination of sequence composition, higher-order structure, protein–protein interaction, and subcellular localization. Reviews of OGT and OGA substrate-selection mechanisms emphasize that enzyme specificity is shaped by more than catalytic activity alone, and that recruitment, accessibility, and multiprotein context are likely to be important determinants of modification [87]. Consistent with this, experimental analysis of OGT glycosylation sites has revealed composition preferences in target sequences, but these preferences are not sufficient to define a robust predictive rule for all substrates [86].

This incomplete understanding has several consequences. At the basic research level, it limits the ability to explain how specific glycosylation events are selected during development, stress adaptation, immune activation, or disease progression. At the translational level, it makes rational targeting more difficult, because broad inhibition of OGT, OGA, or glycosyltransferase families may affect many substrates beyond the one of interest. Without clearer knowledge of how enzymes distinguish among candidate proteins, it remains challenging to move from descriptive glycoproteomics to selective mechanistic intervention.

Some recent tools offer partial ways forward. For example, photoactivatable OGT-based approaches can help capture interaction environments associated with substrate recruitment and may provide insight into how the TPR domain contributes to selective recognition [91]. Similarly, targeted chemical strategies for directing O-GlcNAcylation toward selected proteins suggest that substrate-specific modulation may eventually become experimentally feasible [93]. However, these developments do not yet resolve the broader biological question of how endogenous enzyme systems choose their substrates under physiological conditions.

Overall, incomplete knowledge of substrate selection remains a central obstacle in O-glycosylation research. Identification of modified proteins has advanced rapidly, but the rules that govern enzyme specificity are still only partially defined. Clarifying these rules will be essential if the field is to move from cataloging O-glycosylation events toward explaining their selectivity, predicting their occurrence, and manipulating them with precision [86,87].

### 6.3. Context-Dependent Functional Interpretation Remains Difficult

A third major challenge is that the functional consequences of O-glycosylation are highly context dependent, making biological interpretation difficult even when modification events are clearly detected. The same type of glycosylation change may produce very different outcomes depending on cell type, subcellular localization, metabolic state, disease stage, and the identity of the modified substrate. As a result, it is often not sufficient to conclude that O-glycosylation is simply “protective” or “pathogenic” in a general sense. Its effects must instead be interpreted within a specific biological framework.

This problem is especially evident for O-GlcNAcylation. In some settings, O-GlcNAcylation appears to support host defense or limit tissue damage. For example, O-GlcNAc modification of STING promotes antiviral innate immune signaling, indicating that glycosylation can enhance protective immune responses under infectious stress [80]. Likewise, O-GlcNAcylation of GSDMD has been shown to weaken LPS-induced endothelial pyroptosis, suggesting that this modification can restrain excessive inflammatory injury in certain contexts [94]. These findings indicate that O-glycosylation may function as a buffering mechanism that prevents uncontrolled inflammatory damage.

In other situations, however, the same general modification system is associated with pathological amplification of inflammation or disease progression. GLUT1-mediated O-GlcNAcylation of HMGB1 promotes high-glucose-induced neutrophil extracellular trap formation and aggravates fibroblast inflammation, illustrating how O-GlcNAcylation can also enhance inflammatory pathology under metabolically altered conditions [88]. In heart failure, OGT-mediated O-GlcNAcylation regulates macrophage polarization through IRF1, further supporting the idea that the biological consequence of glycosylation depends on the cellular program into which it is integrated rather than on the modification alone [95].

A similar interpretive difficulty arises in cancer. Increased O-GlcNAcylation can promote tumor-cell survival, therapy resistance, or adaptation to stress, but the specific outcome varies depending on the substrate involved and the form of cellular stress being examined. In glioblastoma and colorectal cancer, inhibition of O-GlcNAcylation can sensitize tumor cells to therapy and alter their mode of cell death [60,62]. Yet even within ferroptosis-related pathways, the direction of effect is not uniform. O-GlcNAcylation can regulate ferroptosis sensitivity through different substrates in different tumor types, as shown for TFRC in hepatocellular carcinoma and METTL3/HMGB1-related regulation in pancreatic cancer [63,64]. These examples show that global statements about the role of O-GlcNAcylation in tumor progression are often too coarse to capture the actual biology.

Mucin-type O-glycosylation presents a related problem. In one setting, dense O-glycosylation of mucins may preserve epithelial barrier function and support tissue homeostasis; in another, altered mucin glycosylation may favor tumor progression, microbial adhesion, or immune escape. For example, intestinal mucin glycosylation can contribute to protection against inflammation and barrier disruption [77,78], whereas abnormal glycosylation of tumor-associated mucins may generate immune-evasive surfaces or expose cancer-associated glyco-epitopes. Thus, even when the molecular substrate class appears similar, the biological meaning of the modification may differ substantially according to disease context.

This context dependence creates a practical challenge for both mechanistic studies and therapeutic translation. If the same glycosylation pathway can either suppress damage or promote pathology depending on the situation, then broad interventions may produce mixed or even contradictory outcomes. For this reason, functional interpretation should move away from global assumptions about “high” or “low” glycosylation and instead focus on defined substrate–pathway–context relationships. In many cases, the critical question is not whether O-glycosylation occurs, but which protein is modified, in which compartment, under which stimulus, and with what downstream consequence.

Overall, the field still faces a major interpretive limitation: O-glycosylation events are increasingly detectable, but their biological meaning often remains conditional rather than absolute. Resolving this problem will require more studies that combine site-specific analysis with tightly defined cellular context and functional perturbation. Until then, context-dependent effects will remain one of the main reasons why O-glycosylation is difficult to translate from descriptive observation into precise mechanistic understanding [80,88,94].

## 7. Conclusions

Overall, the evidence reviewed here supports our hypothesis that mucin-type O-GalNAc glycosylation and O-GlcNAcylation operate through distinct molecular mechanisms but converge on signaling regulation, immune homeostasis, and disease susceptibility. Mucin-type O-glycosylation primarily shapes secreted and cell-surface proteins, thereby influencing receptor modulation, adhesion, barrier integrity, immunoglobulin function, antigen recognition, and tumor–immune communication. In contrast, O-GlcNAcylation acts as a reversible intracellular signaling modification that links nutrient status, stress responses, transcriptional control, and innate immune pathways. These two systems should therefore not be treated as interchangeable, but as complementary regulatory layers.

In disease contexts, aberrant O-linked glycosylation contributes to cancer progression and immune evasion, autoimmune pathology, pathogen–host interactions, and inherited glycosylation defects. Advances in LC–MS/MS-based glycoproteomics, O-glycoprotease-assisted workflows, and spatiotemporal O-GlcNAc profiling have improved detection and mechanistic interpretation. However, major challenges remain in site-specific quantification, glycan structural resolution, enzyme substrate selection, and context-dependent functional validation. Future studies should integrate precise analytical workflows with substrate-specific perturbation and clinically relevant models. Such efforts may help translate O-linked glycosylation biology into glycan-informed biomarkers, patient stratification strategies, and more selective therapeutic approaches.

## Figures and Tables

**Table 1:** Representative genotype–phenotype correlations in glycosylation-related disorders. This table summarizes selected glycosylation-related genes discussed in this review and their associated disease or immune phenotypes. B3GALNT2 and POGLUT1 illustrate O-linked glycosylation-related disorders involving α-dystroglycan glycosylation or Notch O-glucosylation, whereas ALG3 and MAN2A2 are included as representative N-glycosylation-related CDG examples to provide broader genetic context. GALNT14 and C1GALT1C1/Cosmc highlight immune-relevant O-glycosylation mechanisms linked to IgA nephropathy and B-cell tolerance.

Gene	Glycosylation Pathway	Associated Disorder or Phenotype	Molecular Mechanism	Main Clinical or Phenotypic Features	References
B3GALNT2	O-mannosylation/α-dystroglycan glycosylation	B3GALNT2-related dystroglycanopathy	Impaired α-dystroglycan glycosylation reduces functional interaction with extracellular matrix components	Broad phenotypic spectrum, including severe muscle–eye–brain disease, Walker–Warburg syndrome-like presentations, limb-girdle muscular dystrophy, white matter abnormalities, cerebellar hypoplasia, and isolated intellectual disability	[5]
POGLUT1	O-glucosylation of Notch EGF-like repeats	POGLUT1-related muscular dystrophy	Defective O-glucosylation impairs Notch receptor maturation and signaling competence	Muscular dystrophy, reduced Notch signaling, impaired satellite-cell maintenance, and muscle regeneration defects	[21]
ALG3	N-glycan precursor assembly in the ER	ALG3-CDG	Defective ER mannosyltransferase activity disrupts early N-glycan assembly	Multisystem congenital disorder with neurological, ocular, skeletal, dysmorphic, and developmental manifestations	[6]
MAN2A2	N-glycan processing in the Golgi	MAN2A2-related glycosylation defect	Impaired N-glycan processing causes accumulation of immature or hybrid-type serum N-glycans	Autism spectrum disorder, intellectual disability, cognitive delay, and subtle dysmorphic features	[7]
GALNT14	Mucin-type O-GalNAc glycosylation/IgA1 O-glycosylation-related pathway	IgA nephropathy-related glycosylation defect	Altered GalNAc-T14 function links defective O-glycosylation with abnormal IgA biology and B-cell homing	Galactose-deficient IgA1, antiglycan antibody recognition, immune-complex formation, mesangial deposition, and glomerular injury	[69,70]
C1GALT1C1/Cosmc	Core 1 mucin-type O-glycan biosynthesis	B-cell tolerance defect and autoimmune phenotype	Impaired Cosmc-dependent core 1 O-glycan synthesis disrupts normal B-cell glycosylation and tolerance control	Spontaneous autoimmunity and breakdown of B-cell tolerance in experimental models	[43]

Abbreviations: CDG, congenital disorder of glycosylation; EGF, epidermal growth factor; ER, endoplasmic reticulum; IgA1, immunoglobulin A1.

**Table 2:** Analytical strategies for mucin-type O-glycosylation and O-GlcNAcylation analysis. This table summarizes representative analytical approaches used to study mucin-type O-GalNAc glycosylation and O-GlcNAcylation, with emphasis on their major applications, strengths, and limitations. LC–MS/MS-based glycoproteomics is particularly useful for identifying O-glycosylated proteins, assigning glycopeptide compositions, and localizing modification sites, whereas complementary strategies such as O-glycoprotease-assisted workflows, ion mobility, and bioinformatic annotation improve the analysis of densely glycosylated or structurally heterogeneous regions. In contrast, O-GlcNAcylation requires distinct approaches because it is a reversible intracellular modification involving a single GlcNAc residue rather than an extended glycan chain. Antibody-based detection, chemoenzymatic labeling, site-specific mass spectrometry, and OGT/OGA perturbation are therefore commonly used to detect and functionally validate O-GlcNAc-dependent signaling events.

Category	Key Issue	Recent Advances	Remaining Limitations	Future Priorities	Representative References
Site and structural resolution	Accurate localization of O-glycosylation sites and discrimination of heterogeneous glycoforms remain difficult, especially in densely modified peptides.	EThcD-based workflows, ion mobility, and de novo glycopeptide analysis have improved both site assignment and structural interpretation.	Closely spaced glycosylation sites and isomeric glycoforms remain difficult to resolve in complex samples.	More robust site-scoring systems and integrated platforms for isomer-sensitive structural annotation.	[82,83]
Mucin-domain analysis	Highly glycosylated and repetitive mucin-type regions are analytically difficult to digest, fragment, and reconstruct.	O-glycoproteases and middle-down strategies have expanded access to mucin-rich domains.	Domain-level organization of clustered O-glycans is still hard to define comprehensively.	Workflow integration for regional mapping of densely glycosylated extracellular domains.	[82,83]
Quantification	Reliable site-specific quantification of O-glycoforms remains limited.	Comparative glycoproteomics and targeted MS approaches allow relative quantification in selected systems.	Low-abundance glycoforms and workflow variability reduce reproducibility across experiments.	Standardized quantitative pipelines and improved cross-platform comparability.	[82]
Spatiotemporal dynamics	Most available datasets remain static and do not capture when and where glycosylation changes occur.	Live-cell O-GlcNAc tools and spatiotemporal proteomics have improved temporal and compartmental resolution.	Dynamic mapping remains underdeveloped, especially for mucin-type O-glycosylation in the secretory pathway.	Integrated spatial, temporal, and site-resolved approaches for dynamic glycosylation analysis.	[84,85]
Enzyme specificity	Substrate selection by ppGalNAc-transferases, OGT, and related enzymes remains incompletely understood.	Structural and substrate-preference studies have revealed context-dependent recognition features.	No broadly predictive rule currently explains substrate selection across biological contexts.	Mechanistic models integrating sequence, structure, localization, and enzyme recruitment.	[86,87]
Functional interpretation and translation	The biological consequences of O-glycosylation are highly context dependent, which complicates causal interpretation and clinical application.	Functional studies increasingly link O-glycosylation to signaling, immunity, and disease phenotypes, and candidate biomarkers or glycan-targeted strategies are emerging.	Context-dependent effects and limited standardization still restrict mechanistic generalization and translation.	Substrate-specific functional perturbation and validation in clinically relevant systems and patient cohorts.	[36,44,88]

Abbreviations: EThcD, electron-transfer/higher-energy collision dissociation; GalNAc, N-acetylgalactosamine; GlcNAc, N-acetylglucosamine; LC–MS/MS, liquid chromatography–tandem mass spectrometry; MS, mass spectrometry; OGA, O-GlcNAcase; O-GlcNAc, O-linked N-acetylglucosamine; OGT, O-GlcNAc transferase; ppGalNAc-transferases, polypeptide N-acetylgalactosaminyltransferases.

## Data Availability

No new data were created or analyzed in this study.
